# New [3+2+1] Iridium Complexes as Effective Phosphorescent Sensitizers for Efficient Narrowband Saturated–Blue Hyper–OLEDs

**DOI:** 10.1002/advs.202301112

**Published:** 2023-08-31

**Authors:** Chengcheng Wu, Kai‐Ning Tong, Kefei Shi, Zhaoyun Jin, Yuan Wu, Yingxiao Mu, Yanping Huo, Man‐Chung Tang, Chen Yang, Hong Meng, Feiyu Kang, Guodan Wei

**Affiliations:** ^1^ Tsinghua–Berkeley Shenzhen Institute (TBSI) Tsinghua University Shenzhen 518055 China; ^2^ Institute of Materials Research Tsinghua Shenzhen International Graduate School Tsinghua University Shenzhen 518055 China; ^3^ PURI Materials, Inc Shenzhen 518133 China; ^4^ School of Chemical Engineering and Light Industry Guangdong University of Technology Guangzhou 510006 China; ^5^ School of Advanced Materials Shenzhen Graduate School Peking University Shenzhen 518055 China

**Keywords:** blue OLEDs, high efficiency, iridium complex, phosphorescence, sensitizer

## Abstract

Two newly designed and synthesized [3+2+1] iridium complexes through introducing bulky trimethylsiliyl (TMS) groups are doped with a terminal emitter of v–DABNA to form an coincident overlapping spectra between the emission of these two phosphors and the absorption of v–DABNA, creating cascade resonant energy transfer for efficient triplet harvesting. To boost the color quality and efficiency, the fabricated hyper‐OLEDs have been optimized to achieve a high external quantum efficiency of 31.06%, which has been among the highest efficiency results reported for phosphor sensitized saturated‐blue hyper‐OLEDs, and pure blue emission peak at 467 nm with the full width at half maxima (FWHM) as narrow as 18 nm and the CIEy values down to 0.097, satisfying the National Institute of Standards and Technology (NIST) requirement for saturated blue OLEDs display. Surprisingly, such hyper‐OLEDs have obtained the converted lifetime (LT_50_) up to 4552 h at the brightness of 100 cd m^−2^, demonstrating effective Förster resonance energy transfer (FRET) process. Therefore, employing these new bulky TMS substituent [3+2+1] iridium(III) complexes for effective sensitizers can greatly pave the way for further development of high efficiency and stable blue OLEDs in display and lighting applications.

## Graphical abstract

To improve the device stability, efficiency and color purity, such hyper−OLEDs employing the B‐5‐TMS to sensitize the v‐DABNA doped in co‐hosts depicted the converted lifetime (LT_50_) up to 4552 h at the luminance of 100 cd m^−2^ with the enhanced external quantum efficiency (*EQE*_max_)up to 33.42% and full width at half maxima (FWHM) as narrow as 17 nm.

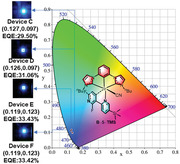

## 1 Introduction

Organic light–emitting diodes (OLEDs) have been pumped into a new era of large–area, transparent, flexible, and energy–saving display and lighting products.^[^ ^1^, ^2^, ^3^, ^4^ ^]^ Both the commercialized emissive materials and efficient OLED devices have consistently achieved successful adoption in academics and industry.^[^ ^5^, ^6^, ^7^, ^8^ ^]^ Nowadays, iridium complexes for red and green emissive layers have been widely employed commercially, however, it remains challenging for saturated blue emitters in terms of color purity, stability, and efficiency.^[^ ^9^, ^10^ ^]^

**Figure 1:**
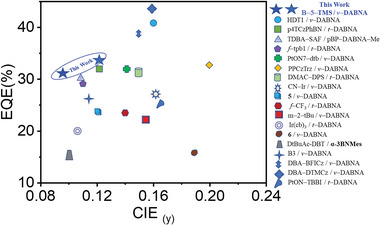
The reported literatures of the BN–based saturated blue–emitting (CIE_y_ < 0.20) hyper–OLEDs.

Very recently, many efforts have been carried out to satisfy the requirement for ideal saturated–blue emissive dopants through phosphorescence and thermal activated decayed fluorescence (TADF) processes, due to their capacities for capturing both the singlet and triplet excitons, achieving the internal quantum efficiency up to unity.^[^ ^11^, ^12^ ^]^ However, the OLEDs based on multiresonance TADF (MR–TADF) materials have been suffering from the aggregation caused quenching (ACQ) process of the emitter due to their planar structure as well as the inefficient triplet exciton utilization for large energy gap between the triplet and singlet levels.^[^ ^13^ ^]^ Moreover, the poor device stability based on the MR–TADF emitter could be attributed to the long–lived excitons having high–energy triplet state.^[^ ^14^, ^15^ ^]^ For phosphorescent OLEDs (PhOLEDs), employing the transitional metal complexes, the emissive dopants have been considered one of the most promising candidates for accelerating the intersystem crossing (ISC) process via spin–orbital coupling. For examples, the iridium based blue emitter featuring the N–heterocyclic carbene(NHC) ligand achieving a saturated blue emission at ≈420 nm with the commission international de I'Eclairage (CIE) coordinates of (0.16, 0.09), corresponding to the external quantum efficiency (*EQE*) up to 10.1%.^[^ ^16^ ^]^ To improve the *EQE*, the iridium emitter based on the phenylpyridine–type has reached an excellent *EQE* of 31.9%, but the CIE coordinates of (0.14, 0.19).^[^ ^17^ ^]^ Moreover, iridium emitter based on the phenylpyrimidine–type exhibited a long operational device lifetime of LT_50_ (the lifetime for the initial luminance down to half) > 2200 h.^[^ ^18^ ^]^ Meanwhile, platinum based emitters have also been developed, in which the tetradentate–type Pt with the planar architecture is beneficial for the delocalization of the electron density, resulting in the narrower emission band to enhance the color purity. These emitters demonstrated sharped vibronic emission band with the full width at half maximum (FWHM) values of ≈20 nm for peak emission between 440 and 450 nm, and one of the devices exhibited the *EQE* value up to 24.8%.^[^ ^19^ ^]^ Subsequently, the exceptionally stable blue PhOLEDs based on the tetradentate–type Pt emitter displayed the longest device operational lifetime at the 1000 cd m^−2^ reported for blue PhOLEDs with CIE y < 0.20.^[^ ^20^ ^]^ On the other hand, the promising candidate for pure blue emitter is multiple–resonance thermal activated delayed fluorescence (MR–TADF), for which achieved the internal quantum efficiency close to 100%, and high color quality of full width at half–maximum (FWHM)<20 nm.^[^ ^21^, ^22^, ^23^ ^]^ However, the current MR–TADF OLEDs have been suffering from the high efficiency roll–off and poor device stability owing to the long excited state lifetime and unbalanced energy transfer in their emissive layer. The long–excited state lifetime of these MR–TADF emitters has caused the inefficient transformation of the triplet excitons into singlet excitons, and unnecessary triplet exciton related efficiency loss such as triplet–triplet annihilation (TTA) and singlet–triplet quenching (STQ). Thus, their device efficiencies become especially deteriorated at high brightness or high current density, which specifically need to be addressed through a short–excited state lifetime or low triplet exciton density. For the same boron–derived MR–TADF emitter such as the well–known *t*–DABNA and *v*–DABNA have been developed to realize phosphor–sensitized TADF devices to reduce the triplet exciton density, which has been so–called hyper–OLEDs.^[^ ^18^ ^]^ Deep blue emitting phosphorescent emitters such as imidazo[4,5–b]pyridin–2–ylidene–based Ir (III), tris–bidentate purin–8–ylindene Ir(III), and Pt(III) complexes are typically introduced as sensitizers. Overall, the fundamental operation mechanism of these unique and efficient hyper–OLEDs could be summarized as followed: i) introducing the bulky groups in sensitizers to maintain the sufficient space to extinguish the circulating triplet excitons via Dexter energy transfer (DET);^[^ ^24^, ^25^ ^]^ ii) boosting the overlapping spectra between the emission of the sensitizer and the absorption of the terminal emitter to promote the Förster resonant energy transfer (FRET) process;^[^ ^26^, ^27^ ^]^ iii) modulation of reasonable energy levels to optimize the sensitizer, emitter, and host to refrain from trapping charges in emissive layer.^[^ ^28^, ^29^, ^30^ ^]^ The fabricated hyper–OLEDs have been demonstrated improved device efficiency and lifetime as shown in **Figure** **1** and Table S1 (Supporting Information). A typical drawback of the devices reported in Table S1 (Supporting Information) was that the critical parameter of CIE_y_ values of these reported hyper–OLEDs has still remained larger than 0.1, and there is a gap to satisfy the national institute of standards and technology (NIST) requirement for saturated blue OLEDs display.

**Scheme 1:**
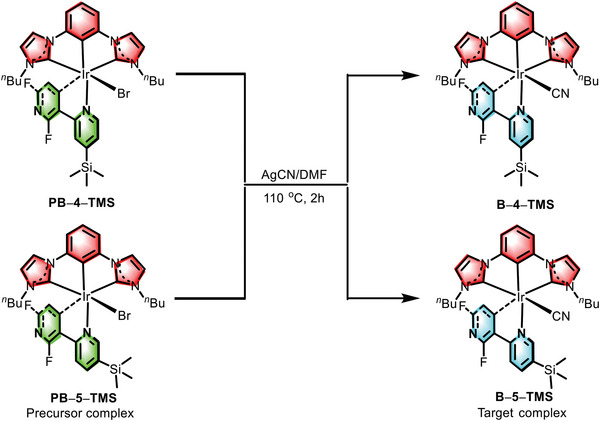
Synthesis of iridium complexes **Β–4–ΤMS** and **Β–5–ΤMS**.

Herein, we have successfully constructed a novel style of [3+2+1] coordinated iridium (III) phosphorescent sensitizers featuring the trimethylsiliyl (TMS) group on the cyclometalated C^N ligand, and the molecular structures of **Β–4–ΤMS** and **Β–5–ΤMS** are illustrated in Scheme 1. To further understand the influence of the bulky TMS substituent on electroluminance (EL) performance in vacuum–deposited OLEDs, their inter/intramolecular interaction in the thin films, photophysical and electrochemical characteristics have been systematically investigated. In this study, complex **B–5–TMS** demonstrates the inter/intra molecular (C–F**^…^** H/C–N**^…^** H) hydrogen bonding in the steady state, as discovered in the X–ray crystal analysis, indicating the existence of an ordered packing model. In addition, the PL characteristics of the **Β–5–TMS** exhibited a vibronic emission peak at ≈445/465 nm, the photoluminescence quantum yield (PLQY) values are close to 90% in solution and the rapid decay time is ≈3 µs in solid thin film. The PL property by solvent–dependent polarity at low temperature indicates that the excited state is charge transfer (CT) characteristic for both complexes. Based upon the advantages of the high internal quantum efficiency and the rapid decay in such obtained iridium complexes, the phosphorescent OLEDs doped with the **Β–5–TMS** have exhibited the *EQE*_max_ and *CE*_max_ of 27.77% and 37.05 cd A^−1^. In addition, given the sufficient distance by introducing the bulky TMS substituent on the iridium complexes, the rational molecular orbital levels in host, as well as the overlapping spectra of their emission of the **Β–5–TMS** phosphor and the absorption of v–DABNA, the hyper–OLEDs have demonstrated the efficient energy transfer via FRET, in which the sensitized devices exhibited the *EQE* as high as 31.06% and relative emission peak at 467 nm with the FWHM as narrow as 18 nm and the CIE_y_ values down to 0.097, satisfying the NIST requirement for saturated blue OLEDs display. With an intention to further improve the device stability, we fabricated blue OLED devices to conduct the operational lifetime. To note, such hyper–OLEDs depicted the converted lifetime (LT_50_) up to 4552 h at the brightness of 100 cd m^−2^ with the enhanced *EQE*_max_ up to 33.42%, which has been among the highest efficiency results reported for phosphor sensitized saturated blue hyper–OLEDs, confirming the advantages of these two bulky TMS substituented Ir(III) complexes for effective sensitizers. This work will greatly pave the way for further development of blue OLEDs in display and lighting applications.

## 2 Results and Discussion

### 2.1 Molecular Design and Characterization

**Figure 2:**
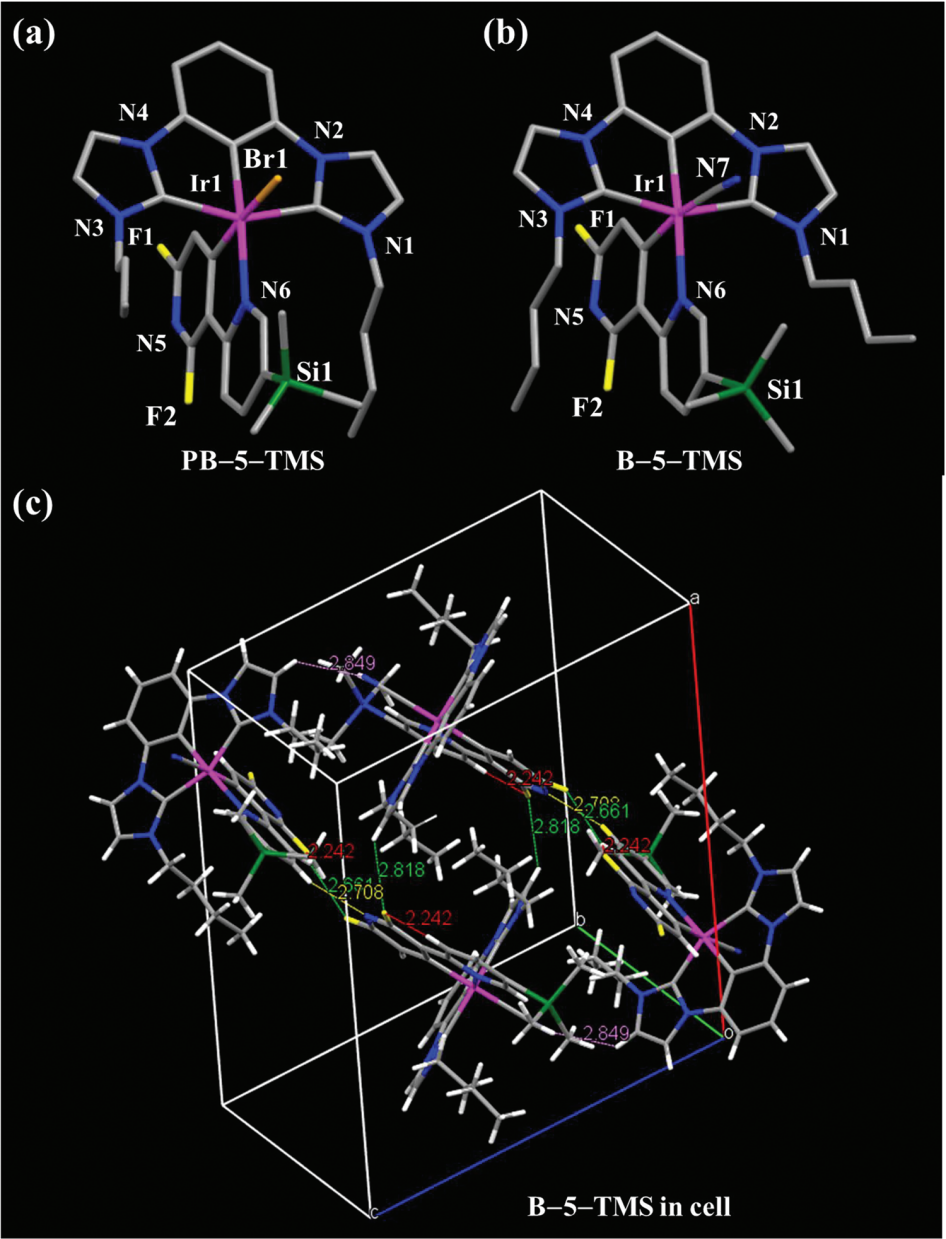
Perspective views of a) **PB–5–TMS** and b) **B–5–TMS**. Hydrogen atoms and solvent molecules are omitted for clarity. c) The 1D supramolecular network of **B–5–TMS** formed intramolecular (red dashed lines, 2.242 Å) and intermolecular interactions (green dashed lines, 2.661 and 2.818 Å; yellow dash lines, 2.708 Å; and violet dash lines, 2.849 Å).

The syntheses and relative structures of the iridium complexes are depicted in **Scheme** **1**. The target complexes **B–4–TMS** and **B–5–TMS** were obtained from the reaction of the corresponding bromide intermediates (**PB–4–TMS** and **PB–5–TMS**) with silver cyanide (AgCN) in dimethyl formamide (DMF) solution. The precursors and the ligands were prepared according to the literature,^[^ ^17^, ^31^, ^32^, ^33^, ^34^ ^]^ and all the compounds were characterized by ^1^H NMR and ^19^F NMR spectroscopy and mass spectrometry (see the Experimental Section Figures S1–S18, Supporting Information). The thermal stability of the iridium complexes was studied by thermogravimetric analysis (TGA) and differential scanning calorimetry (DSC) (Figures S19 and S20 and Table S2, Supporting Information). The iridium complexes reveal high thermal decomposition temperatures (T_d_, defined as 5% weight loss) approach to 350 °C, indicating that both **B–4–TMS** and **B–5–TMS** were suitable for OLED preparation by thermally vacuum deposition. The introduction of trimethyl silicon (**TMS**) groups at C^N ligands of blue emitters could introduce highly twisted crystal structure through mitigating the π–π interactions of these molecules in the steady state, as evidenced by the X‐ray crystal analysis in **Figure** **2**.

### 2.2 Crystallography

As depicted in Figure 2, the molecular structures of **PB–5–ΤΜS** and **B–5–ΤΜS** are similar except for the monodentate ligand. The six–coordinated architecture surrounded by three C atoms from *C^C^C* ligand and one C atom and one N atom from *C^N* ligands, as well as one monodentate ligand (**Br** or **CN**)—forming a distorted octahedral geometry in metal central. The physical parameters of the two single crystals are close to each other, and the details reported in Table S3 (Supporting Information). Specially, the monodentate bond length of Ir–Br (*av*. Ir–Br 2.6039(4) Å) in **PB–5–ΤΜS** are slightly longer than those (*av*. Ir–C34 2.039(4) Å) in **B–5–ΤΜS**, which could be attributed to the longer radius of the bromine atom when compared to the carbon atoms, consistent with the reported work.^[^ ^35^, ^36^ ^]^ Interestingly, the length of Ir–C21 (*av*. 2.030(4) Å) in **B–5–ΤΜS** is slightly longer than those (*av*. 1.978(3) Å) in **PB–5–ΤΜS**, which could be caused by the stronger field ligand of **CN** through the inductive effect. In the 1D supramolecular network of the **B–5–ΤΜS**, the intramolecular C–H**^…^** F (red line, 2.242 Å) and intermolecular interactions (green dashed lines, 2.661 and 2.818 Å; yellow dash lines, 2.708 Å; and violet dash lines, 2.849 Å) have been obtained in the Figure 2. The dimer–like structure exists inter/intramolecular interactions, such as hydrogen bond, which might be to the benefit of charge transfer in the molecular network.^[^ ^37^, ^38^ ^]^

### 2.3 Photophysical Properties

**Figure 3:**
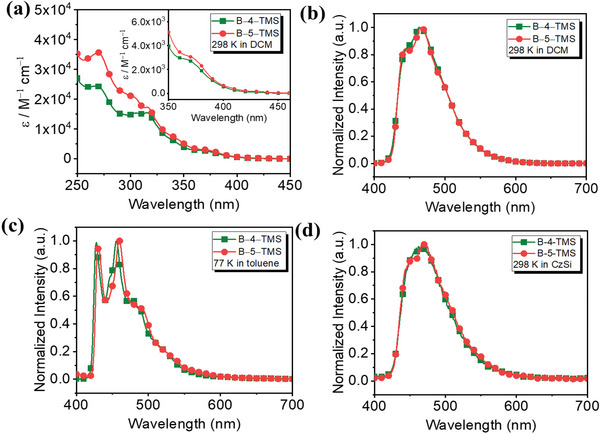
a) Absorption (magnified absorption spectra) of B–4–TMS and B–5–TMS complexes in dichloromethane (DCM) solution at 298 K; b) Emission spectra in B–4–TMS and B–5–TMS complexes in DCM (*c* = 2 × 10^−5^ m) solution at 298 K; c) Emission spectra in toluene at 77 K; d) Emission spectra in 12.5 wt.% doped CzSi film.

To investigate the photophysical characteristics, the UV–vis absorptions and the normalized photoluminescence (PL) spectra of the iridium complexes recorded in dichloromethane (DCM) as displayed in **Figure** **3**, and the data are recorded in **Table** **1**. The intense absorption bands with extinction coefficients in the order of 10^4^ m ^−1^ cm^−1^ around at 270 nm were assigned to ligand centered (C^C^C or C^N) transitions with spin–allowed π–π* character (^1^IL, π–π*). The moderately intensity absorption shoulders in the 320 nm (1 × 10^4^ < *ε* <2 × 10^4^ m ^−1^ cm^−1^) observed for all complexes were assigned to spin–allowed singlet ligand–centered/ligand–to–ligand charge transfer (^1^LC/^1^LLCT) transitions; In addition, the broad absorption band ≈350–400 nm can be assigned to spin allowed metal–ligand charge–transfer (^1^MLCT) bands with extinction coefficients in the order of 10^3^ m ^−1^ cm^−1^. Furthermore, the spin–forbidden ^3^MLCT transition bands weakly observed ≈400 to 450 nm. According to the previously reported, intense MLCT bands implied that the origin might be predominant from metal d orbitals.^[^ ^31^ ^]^ In contrast, the weak MLCT band had its origins in a poor contribution of metal d orbitals. Complex **B–5–ΤΜS** had a slightly stronger absorption with the extinction coefficient (*ε* ≈3300 m ^−1^ cm^−1^) than that of **B–4–ΤΜS** (*ε* ≈2600 m ^−1^ cm^−1^) in the ^1^MLCT area. This result indicated that complex **B–5–TMS** has the greater contribution from iridiu *d* orbitals, corresponding to those of **B–4–ΤΜS,** which could be consistent with the computation part.

**Table 1:** Photophysical properties of iridium (III) complexes

Complex	Absorption ^a)^	Emission, 298 K Sol.			Emission, 77 K Sol.		Emission, film		
	*ε* x 10^4^ m ^−1^ cm^−1^	λ_max_ ^b)^[nm]	𝝫_PL_ ^c)^[%]	τ [µs]	λ_max_ ^d)^[nm]	*T*_1_ [eV]	λ_max_ ^e)^[nm]	𝝫_PL_ ^c)^ [%]	τ [µs]
**B–4–TMS**	270(3.59), 316(1.75), 370(0.33)	447,463	87	0.42	428,456	2.90	467	70	2.9
**B–5–TMS**	270(2.43), 316(1.55), 371(0.26)	443,467	88	0.36	430,460	2.88	470	75	2.7

^a,b)^ Absorption and emission spectrum was measured in toluene; [m] = 2.0 × 10^−5^;

^c)^ Phosphorescence quantum efficiency measured by absolute method using integrating sphere;

^d)^ Emission spectrum was measured in toluene; λexc = 320 nm.

^e)^ Emission spectrum was measured in 12.5 wt.% doped CzSi film, *λ*_exc_ = 320 nm.

As depicted in the Figure 3b–d, the normalized emission characters of **B–4–ΤΜS** and **B–5–ΤΜS** have significant similarities, and both them have the peak emission at ≈465 nm with a shoulder peak at ≈445 nm at room temperature in toluene; but the emission of the **B–5–ΤΜS** exhibited a slightly red–shift at the low temperature and solid state, which could be attributed to the less inductive effect at the meta–position rather than para–position of the TMS group. Upon the temperature down to 77 K, the normalized emission of the two emitters displayed a vibronic structure with a 20 nm blue shift, and the shoulder peak split to a sharp peak. Considering the effect of temperature on the excited state, the triplet energies (E_T_) were obtained for **Β–4–TMS** is 2.80 eV and **Β–5–TMS** is 2.78 eV at 77 K, respectively, which is slightly higher than (dfpy–4–tms–py)_2_Ir(acac) and (dfpy–5–tms–py)_2_Ir(acac).^[^ ^31^ ^]^ Such a phenomenon indicated that the configurations of the [3+2+1] iridium complexes are more conductive for fabricating the saturated blue device with higher triplet energy of the emitters by using the same C^N main ligand. To confirm the excited state nature of the charge transfer (CT) for **Β–4–TMS** and **Β–5–TMS**, the emission characteristics of the solvent–dependent are recorded in Figure S24 (Supporting Information). **Β–4–TMS** and **Β–5–TMS** displayed the similar behaviors with the polarity of the solvents increased from the toluene to *N, N*–dimethylformamide (DMF), a discrepancy of the emission from the vibronic structure to the gaussian distribution was observed, which could be contributed to the LLCT band are more sensitive to the polarity of the solvent, resulting in an increased CT nature of the phosphors.

Both the complexes shown high internal quantum efficiencies of up 0.70 and 0.75 in solid state at doped 9–(4–tert–Butylphenyl)–3,6–bis(triphenylsilyl)–9H–carbazole (CzSi) film (see Table 1). Additionally, the transient PL features of **Β–4–TMS** and **Β–5–TMS** are observed for the excited state lifetimes (τ_obs_), in which the measured decay time of 2.9 µs for **Β–4–TMS** and 2.7 µs for **Β–5–TMS**, respectively, corresponding to the radiative decay rate (*k*_r_) are 2.59 × 10^5^ and 2.78 × 10^5^ s^−1^, respectively, which were higher than the nonsubstituted complexes of dfpypy–CN (*k*_r_ = 1.60 × 10^5^ s^−1^).^[^ ^36^ ^]^ Such a discrepancy implied that the incorporation of the TMS group on the dfpypy ligands have the potential to decrease the TTA and STQ process by enhancing the steric hindrance and accelerating the radiative decay in the emissive layer. The *k*_r_ value of **Β–5–TMS** is greater than that of **Β–4–TMS** because it is inversely proportional to triplet excited state lifetime and higher internal quantum yield. Interestingly, the PL performance that introduction of the TMS group on the 5–position of the pyridine fragment of the C^N ligand is superior to the 4–position. The photophysical properties of PLQY and phosphorescent lifetime for **Β–4–TMS**  or **Β–5–TMS**  were different behaviors in toluene solution relative to the performance in solid state. (Table 1) Both emitters exhibited higher PLQY and shorter excited state lifetime in solution compared with those in CzSi film, which could be contributed to the perturbation of the polarization of the solvent and the interaction between the molecules, resulting in TTA or STQ.^[^ ^39^, ^40^, ^41^, ^42^ ^]^

### 2.4 Electrochemistry

In order to investigate the electrochemical nature, all the complexes were employed in the cyclic voltammetry (CV) experiments to measure the HOMO levels in degassed dichloromethane (0.1 m *^n^* Bu_4_NPF_6_). The CV curves and the detail data are summarized in Figures S25 and S26 (Supporting Information), Table S5 (Supporting Information) and **Table** **2**. All the complexes depicted quasi–reversible oxidation curves, and the E_ox(onset)_ value of 1.21 V and 1.20 V for **Β–4–ΤΜS** and **Β–5–ΤΜS**, respectively, which are lower than that of the non–substituted counterparts dfpypy–CN (E_ox_ = 1.25 V). These results indicated the higher E_ox(onset)_ values than those of classical (dfpypy)_2_Ir(L^X), such as (dfpy–4–tms–py)_2_Ir(acac) (1.03 eV) and (dfpy–5–tms–py)_2_Ir(acac) (0.90 eV), where acac is acetoneacenate.^[^ ^31^ ^]^ According to the oxidation potential and redox couple of Fc/Fc^+^ (4.8 eV below the vacuum level), the highest occupied molecular orbital (HOMO) levels for **Β–4–ΤΜS** and **Β–5–ΤΜS** were estimated to be –5.49 eV and –5.58 eV, respectively. As compared to HOMO level of dfpypy–CN (E_HOMO_ = –5.57 eV), both complexes show shallower HOMO energy levels with a difference of 0.01–0.08 eV. Based on the results that the energy gap obtained from the onset of the absorption, the corresponding lowest unoccupied molecular orbital (LUMO) levels were estimated from the subtraction between the HOMO level and the energy gap, which were found to be –2.35 eV for **Β–4–ΤΜS** and –2.46 eV for **Β–5–ΤΜS**. Overall, the incorporated TMS substituent at the 4/5–position of the pyridine fragment on complexes **Β–4–ΤΜS**  and **Β–5–ΤΜS** has a significant perturbation on the HOMO and LUMO levels, and could promote the larger energy levels compared to nonsubstituted counterparts dfpypy–CN (*E_g_* = 1.07 eV).^[^ ^36^ ^]^

**Table 2:** Experimental HOMO–LUMO energy and TD–DFT calculation results

Complex	Experimental				DFT/TD–DFT result				
	E_ox_ ^a)^ [V]	HOMO^b)^ [eV]	LUMO^c)^ [eV]	E_g_ ^d)^ [eV]	HOMOe) [eV]	LUMO^e)^ [eV]	HOMO–LUMO^e)^ [eV]	%H→L [S_0_→S_1_]	Assignment^f)^
**Β–4–ΤΜS**	1.21	–5.49	–2.35	3.14	–5.56	–1.61	3.95	98	MLCT(29.24%) LLCT/ILCT
**Β–5–ΤΜS**	1.20	–5.58	–2.46	3.12	–5.56	–1.61	3.95	98	MLCT(29.24%) LLCT/ILCT

^a)^ The redox potential of all compounds were measured in CH_2_Cl_2_ with 0.1 m *^n^* Bu_4_NPF_6_ at a scan rate of either 50 or 100 mVs^−1^(versus F_c_^0^/F_c_^+^);

^b)^ E_HOMO_ levels were calculated from electrochemical potentials, i.e., E_HOMO_ = –e(E_pa_+(4.8–F_c_^+^/F_c_)) or E_HOMO_ = –e(E_1/2_^Ox^+(4.8–F_c_^+^/F_c_)). (F_c_^+^/F_c_ = 0.46 V in CH_2_Cl_2_);

^c)^ E_LUMO_ = E_HOMO_ + E*_g_* (where *E_g_* refers to the onset of absorption);

^d)^ Derived from E(Ox)_onset_ – E(Re)_onset_;

^e)^ The theoretical energy levels were calculated from the optimized structure at the ground state;

^f)^ The assignment of MLCT character was calculated from the differences of the metal contribution in the dominant electronic transition at the triplet state.

### 2.5 Computational Studies

To further realize the properties of the absorption and emission, density functional theory (DFT) and time–dependent density functional theory (TD–DFT) simulation were conducted on these two iridium compounds at the PBE0 level of theory. As depicted in the Figures S27 and S28 (Supporting Information), the simulated results of absorption spectra are for the **Β–4–TMS** and **Β–5–TMS**, and the details of the first fifteen singlet excited transition and the relative oscillator strength are recorded in Table S6 (Supporting Information), involving the molecular orbital are shown in Figures S29 and S30 (Supporting Information). The low–energy band of absorption located at ≈327 nm are originated from HOMO–2 → LUMO excitation. The HOMO–2 is the π orbitals localized predominantly on the metal center and the C^N ligand with some localized on monodentate ligand, whereas the LUMO depicted that the π∗ orbital almost entirely localized on the moiety of the C^N ligand. Such an absorption band could be assigned as the mixture contribution of the ^1^MLCT [dπ(Ir)→π*(C^N)] transition and the ^1^ILCT[π(C^N )→π*(C^N)] with some LLCT[π(C^C^C )→π*(C^N), π(CN )→π*(C^N)]. The moderate energy absorption band calculated at ≈297 nm originated from the HOMO–3 → LUMO, HOMO–1 → LUMO+1 and HOMO–2 → LUMO+1excitation, in which are mainly attributed to the LLCT[π(C^C^C )→π*(C^N) and ILCT[π(C^N )→π*(C^N)]. As for the strongest energy band, the simulated the absorption are ≈275 nm are predominantly assigned as the ^1^ILCT[π→π*] transition in the C^N ligand by the HOMO–2 → LUMO+1. Such a result is in good agreement with the experimental absorption spectra.

**Figure 4:**
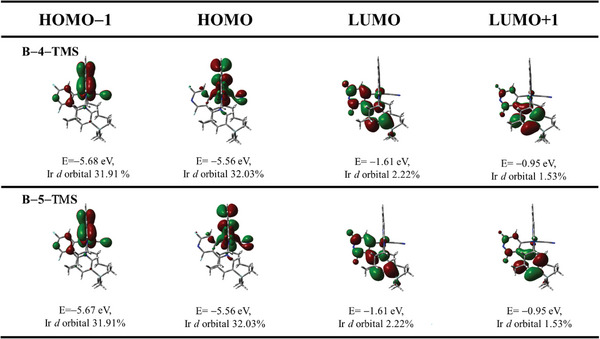
The energy levels and metal contribution of frontier molecular orbitals for the [3+2+1] iridium complexes at their geometries optimized for the ground state.

The orbital energy level diagram of the frontier molecular orbitals for **Β–4–TMS** and **Β–5–TMS** are displayed in the **Figure** **4** and the data summarized in Table 2. By introducing the TMS group on the C^N ligand, the HOMO energy level barely shows differences. In general, the HOMO predominantly localized on the C^C^C ligand, corresponding to the LUMO localized on the C^N ligand, are consistent with the previous work.^[^ ^35^, ^36^ ^]^ By introducing the TMS substituent on the C^N fragment, the calculated energy gaps for these two complexes are identical, and it's consistent with the experimental results, in which the deviation is almost negligible with the difference of 0.02 eV. Nevertheless, the HOMO predominantly localized on the C^C^C ligand, corresponding the LUMO localized on the C^N ligand.

Upon the nature of the emissive state of **Β–4–TMS** and **Β–5–TMS**, the optimized geometries of the lowest–lying triplet state (T_1_) were employing the PBE0/PCM (CH_2_Cl_2_) method, and the details summarized in Table S7 and Figures S31 and S32 (Supporting Information). According to the spatial plots of selected molecular orbitals, the optimized T1 state are mainly originated from the HOMO–2→LUMO and HOMO–1→LUMO excitation, which are predominantly contributed to the ^3^LLCT and mixtured some ^3^MLCT characters, as evidenced by the solvent–dependent PL experiment and low temperature PL experiment. The plots of spin density of the T_1_ states of **Β–4–TMS** and **Β–5–TMS** are basically localized on the dfpypy ligand and partially localized on the Ir center, as depicted in Figure S33 (Supporting Information).

### 2.6 OLED Fabrication

**Figure 5:**
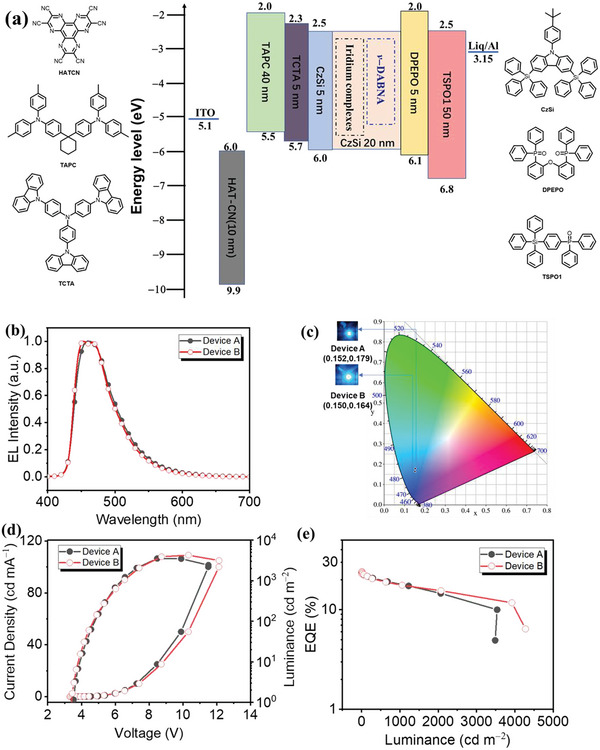
a) The device architecture with the energy levels and the molecular structure in organic layers; b) the EL spectrum; c) CIE coordinates and the images of the devices; d) the *J–V–L* curves; e) the EQE versus luminance. The terminal emitter and the host materials in emissive layer as followed:**Β–4–TMS** (12.5%) doped in CzSi for device A, **Β–5–TMS** (12.5%) doped in CzSi for device B, respectively.

Taking the advantages of the promising photophysical properties for the [3+2+1] coordinated iridium complexes, **B–4–ΤΜS** and **B–5–ΤΜS** are employed to investigate their electroluminescence (EL) performance. The multilayer devices based on these two newly designed iridium complexes have been fabricated with the device architectures: Indium tin oxide (ITO) / 2,3,6,7,10,11–Hexacyano–1,4,5,8,9,12–hexaazatriphenylene (HATCN, 10 nm)/N–(4–(1–(4–(di–p–tolylamino)phenyl)cyclohexyl)phenyl)–3–methyl–N–(p–tolyl)aniline (TAPC, 40 nm) / 4,4′,4′'–Tri–9–carbazolyltriphenylamine (TCTA, 5 nm) / 9–(4–tert–Butylphenyl)–3,6–bis(triphenylsilyl)–9H–carbazole (CzSi, 5 nm) / x wt.% phosphor (20 nm) doped in CzSi / Bis[2–((oxo)diphenylphosphino)phenyl] ether (DPEPO, 5 nm) / Diphenyl[4–(triphenylsilyl)phenyl]phosphine oxide (TSPO1, 50 nm) / 8–Hydroxyquinolinolato–lithium (Liq, 2 nm) / Aluminum (Al, 120 nm). The energy level diagram and the molecular structures in device are depicted in **Figure** **5**a.

As displayed in Figure S34 (Supporting Information) and Table S10 (Supporting Information), the optimized doping concentrations of 12.5% for the phosphorescent OLEDs based on the iridium complexes as emitters. The phosphorescent device A performance for the **B–4–ΤΜS** dopant exhibited a structureless emission with the peak at ≈460 nm (Figure 5b) and CIE coordinates of (0.152, 0.179) (Figure 5c), while the EL of device B based on the **Β–5–TMS** dopant shown a vibronic emission with the peak at ≈453 and 461 nm, corresponding the CIE coordinates of (0.150, 0.164). Such a minor discrepancy could be contributed to the **Β–4–TMS** are more sensitive to the changed surroundings, such as polarity of the host materials and molecular stacking model in steady state, which is in an agreement with the PL performance, causing by the CT characteristic of the iridium complexes. Moreover, the maximum current efficiency (CE) and power efficiency (PE) have been achieved for device A of 34.91 cd A^−1^, 29.78 lm W^−1^ and for device B of 37.05 cd A^−1^, 32.99 lm W^−1^, respectively, corresponding to the maximum *EQE* up to 25.49% and 27.77% (Figure 5c,d and **Table** **3**). However, the observed EL efficiency is lower for device A, which could be caused by the lower PL efficiency for **B–4–ΤΜS** compared to those of the **B–5–TMS** doped in CzSi host.

**Table 3:** Characteristics of the tested devices

Device^a)^	λ_EL_ [nm]	*L*_max_ [cd m^−2^]	EQE^b)^ [%]	Power efficiency^b)^ [lm W^−1^]	Current efficiency^b)^ [cd A^−1^]	CIE [x,y]	LT_50_ [h]	FWHM [nm]
Control I	469	3489	18.58/–/–	11.33/–/–	14.85/–/–	(0.128, 0.097)	–	18
Control II	470	32 741	19.80/18.23/16.06	18.19/15.47/10.06	17.97/17.51/15.45	(0.125,0.108)	1946	17
Device A	463	4829	25.49/23.85/19.91	29.78/23.43/13.99	34.91/32.67/27.28	(0.152,0.179)	4	64
Device B	461	4471	27.77/22.86/18.91	32.99/22.10/12.78	37.05/30.50/25.23	(0.150,0.164)	8	62
Device C	467	6083	29.50/24.49/20.63	19.71/12.45/8.05	24.77/20.52/17.29	(0.127,0.097)	2	18
Device D	467	8137	31.06/27.43/22.74	21.59/15.50/9.75	26.91/23.76/19.70	(0.126,0.098)	3	18
Device E	470	54 138	33.43/27.27/22.96	34.45/20.16/12.32	31.06/25.33/21.32	(0.119,0.123)	4465	17
Device F	470	53 037	33.42/28.73/24.09	33.21/20.64/12.60	30.79/25.35/21.25	(0.119,0.123)	4552	17

^a)^ The terminal emitter and the host materials in emissive layer as followed: **Β–4–TMS** (12.5%) and CzSi for device A, **Β–5–TMS** (12.5%) and CzSi for device B, *v*–DABNA (1%), **Β–4–TMS** (12.5%) and CzSi for device C, *v*–DABNA (1%), **Β–5–TMS** (12.5%) and CzSi for device D, *v*–DABNA (1%), **Β–4–TMS** (12.5%) and SiCzCz:SiTrzCz2 for device E and *v*–DABNA (1%), **Β–5–TMS** (12.5%) and SiCzCz:SiTrzCz2 for device F, respectively;

^b)^ The value estimated at maximum, 100 cd m^−2^ and 1000 cd m^−2^.

Recently, multi–resonance thermally activated delayed fluorescence (MR–TADF) emitters have been attracted wide attention due to their excellent PLQY, narrow emission FWHM and high color purity.^[^ ^2^, ^22^, ^43^, ^44^, ^45^ ^]^ Giving the large overlapping region between the emission of the iridium complex and the absorption of the emitter, *v*–DABNA are optimized as the terminal emitter due to their particular features of saturated narrowband emission with high efficiency.^[^ ^22^ ^]^ Considering the energy transfer via the FRET process, the triplet energy(T_1_) of the phosphorescent sensitizers is ≈2.88 eV more than the energy level of *v*–DABNA (*S*_1_ = 2.64 eV, *T*_1_ = 2.62 eV), indicating the balanced generation of the saturated–blue hyper–luminescence as realized in this system.

**Figure 6:**
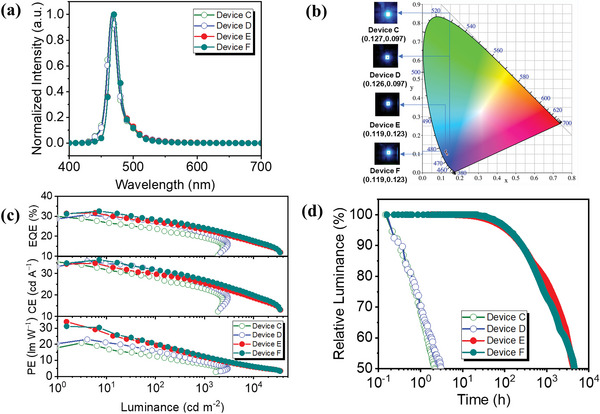
a) the EL spectrum; b) CIE coordinates and the images of the devices. c) EQE, CE, and PE versus luminance; d) device lifetime curves at 100 cd m^−2^. The terminal emitter and the host materials in emissive layer as followed: *v*–DABNA (1%) and **Β–4–TMS** (12.5%) doped in CzSi for device C, *v*–DABNA (1%) and **Β–5–TMS** (12.5%) doped in CzSi for device D; *v*–DABNA (1%), and **Β–4–TMS** (12.5%) doped in SiCzCz:SiTrzCz2 for device E; *v*–DABNA (1%) and **Β–5–TMS** (12.5%) doped in SiCzCz:SiTrzCz2 for device F, respectively.

Encouraged by the successful embodiments of Ph–OLEDs,^[^ ^42^, ^46^, ^48^, ^49^ ^]^ the hyper–OLEDs with similar architectures to the Ph–OLEDs have been extensively carried out. The optimized concentration of the sensitizer of 12.5 wt.%, and the MR–TADF emitter modulated concentration range of 0.5 to 1 wt.% have been investigated, as shown in Figure S36 (Supporting Information). The device performance based on the phosphor sensitized MR–TADF emitter were summarized in Table 3, and **Figure** **6**. As depicted in Figure 6a,b, both the sensitized EL performance of device C and D exhibited a maximum peak at ≈470 nm with the FWHM of 18 nm at ratio of 1.0 wt.%, which are almost identical to the EL performance of *v*–DABNA emitter–only (control I) with the same CIE_y_ coordination of 0.097, satisfying the requirement of National Institute of Standards and Technology (NIST) for saturated–blue OLEDs display application.^[^ ^50^ ^]^ In addition, the hyper–luminescent devices C and D shown increased *EQE*_max_ = 29.50% and 31.06%, respectively, compared to the phosphorescent OLED (25.49% for device A and 27.77% for device B, respectively) and 1.0% *v*–DABNA–only OLEDs (18.58% for control I) (see Figure 6c). Besides, the brightness of the sensitized device C and D are higher than the phosphorescent OLEDs and *v*–DABNA–only OLEDs. Therefore, all the hyper–OLEDs exhibited a higher maximum *EQE* and luminance than those of non–sensitized devices, indicating its greater exciton utilization and efficient energy transfer. In other words, the BN–based emitters without sensitizer are inefficient in trapping the triplet excitons, and the triplet excitons cannot be effectively transferred to the singlet excited state by the reverse intersystem crossing (RISC) process, which could be attributed to the large energy level difference between singlet and triplet energy level, causing accumulated and quenched triple excitons.^[^ ^46^ ^]^ Conversely, the sensitizer provides a rapid channel to transfer the triplet excitons to the singlet excited state in the emissive layer of hyper–OLEDs.^[^ ^47^ ^]^ Moreover, the fast process of the RISC through the FRET by sensitizer has a significant effect on elevating the exciton utilization and enhancing the device performance.^[^ ^51^ ^]^

Further investigation of the high performance saturated–blue OLED based on the [3+2+1] iridium complexes as sensitizer was confirmed by the operating lifetime analysis. However, the operating lifetime of the devices C and D were too short to obtain due to the poor stability of the DPEPO employing as the hole blocking layer.^[^ ^44^ ^]^ Considering this concept, the operating lifetime device has been fabricated, as the device structure following the reported literature,^[^ ^20^ ^]^ due to their balanced charge injection and transporting mobilities. Their EL characteristics summarized in the Table 3, Figure 6, and Figures S35–S37 (Supporting Information), the devices E and F exhibit slightly narrower FWHM than the device C and D. As a result, the CIE coordination of devices E is the same as device F, which are recorded as (0.119, 0.123). The control II device for the *v*–DABNA–only emitter in this structure measured the CIE coordinate of (0.125, 0.108). The significant improvements are been recorded in the CE_max_ (31.06 cd A^−1^for device E and 31.07 cd A^−1^ for device F), PE_max_ (34.45 lmW^−1^ for device E and 33.21 lm W^−1^ for device F), corresponding the *EQE*_max_ up to 33.43% and 33.42%, respectively. Such a promotion could be contributed to the larger recombination and more balanced charge transfer, as evidenced by the thicker emissive layer and the shorten driving voltage. The operational lifetime of LT_50_ (initial brightness drop to half) based on the device E and F at *J* = 10 mA cm^−2^ for the ν–DABNA hyper–OLEDs are more than 20 h (the initial luminance at 2000 cd m^−2^). Converting the device lifetime at 100 cd m^−2^ were 4465 and 4552 h for the devices E and F, respectively, by assuming an acceleration factor of 1.8, as shown in Table 3 and Figure 6d.

**Figure 7:**
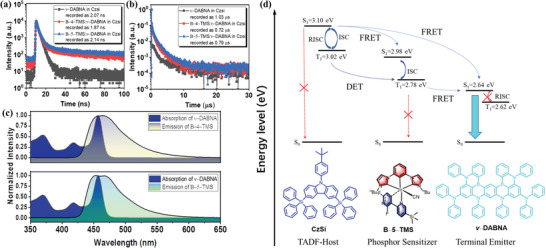
a) Short time scale component of the transient PL decay time in solid state; b) long time scale component of the transient PL decay in solid state; c) spectral overlap region between phosphorescent sensitizer PL and absorption spectra of ν–DABNA emitters in toluene (2 × 10^−5^ m solution); d) proposed energy transfer mechanism in the sensitized system.

To confirm the effective energy transfer process, the transient PL property of the doped films 1% *v*–DABNA in CzSi, 12.5% **B–4–TMS**: 1% *v*–DABNA in CzSi and 12.5% **B–5–TMS**: 1% *v*–DABNA in CzSi are investigated. The excited–state decay of the short time scale for the sensitized films recorded are 1.87 ns and 2.14 ns, respectively, comparable to the *v*–DABNA in CzSi (2.07 ns) (recorded from **Figure** **7**a), which could be originated from the terminal emission of *v*–DABNA from the singlet state to the ground state. As depicted in the Figure 7b, the decay lifetime of the films in long–time scale related to the triplet excitons of the phosphors and *v*–DABNA. The excited state lifetime of **B–4–TMS** and **B–5–TMS** through triplet excitons radiation are 2.88 µs and 2.66 µs, respectively, corresponding to the delayed fluorescence lifetime of *ν*–DABNA via RISC process is 1.03 µs. It is worth noting that the decay time of the two emitter co–doped films in CzSi are 0.72 µs for **B–4–TMS** sensitized *v*–DABNA, 0.79 µs for **B–5–TMS** sensitized *v*–DABNA, respectively, which showed even faster decay than the sole phosphors and *v*–DABNA doped films. Such results indicated that the efficient triplet exciton energy transfer via Förster energy transfer to the *v*–DABNA emitter as well as the almost negligible RISC process from the triplet state to the singlet state in *v*–DABNA. As shown in Figure 7c, the overlapping region that the emission of the phosphors almost covered the absorption of the *v*–DABNA, providing a perfect material combination system of the phosphor sensitizer and TADF emitter for efficient energy transfer. Therefore, the proposed energy transfer mechanism has been demonstrated, as shown in Figure 7d.

Such hyper–OLEDs employed the [3+2+1] coordinated iridium complexes as the efficient sensitizer were designed and fabricated, realized in high color quality, high external efficiency, and good stability. The phosphorescent iridium complexes **Β–4–ΤMS** and **Β–5–ΤMS** featuring the following merits: (i) the incorporated bulky TMS substituent on the C^N ligand have increased the steric hindrance, significantly avoiding the exciton quenching processes; (ii) their rapid excited–state decay processes to reduce the non–radiative recombination; (iii) the almost coincident spectra between the emission of phosphors and the absorption of *v*–DABNA is beneficial for efficient FRET process, meanwhile, (iv) the reasonable HOMO–LUMO levels of the phosphors have shortened the energy gap between the host and *v*–DABNA, which could significantly decrease the population of triplet excitons and polarons via directly trapping, and the triplet excitons cannot be effectively transferred to the singlet excited state by the RISC process.

## 3 Conclusion

By introducing the trimethylsiliyl (TMS) groups on the 2′,6′–difluoro–2′,3′–bipyridine (dfpypy) main ligand, two promising [3+2+1] coordinated saturated–blue emitting iridium complexes are designed and synthesized to investigate their structural, photophysical, and electrochemical properties. The existence of the inter/intramolecular interactions (C–F**…**H/C–N**…**H) is considered as an effective path to accelerate energy transfer. Both the complex **Β–4–TMS** and **Β–5–TMS** functionalized the TMS on dfpypy ligand exhibited PLQY close to 90%, and the vibronic emission peak at 443/465 nm with rapid decay down to 0.36 µs in toluene solution, emerging high radiative decay (*K_r_*) of up to 2.5 × 106 s–1. The pristine phosphorescent OLEDs based on these two phosphors of **Β–4–TMS** and **Β–5–TMS** exhibited the *EQE_max_* of 25.49% and 27.77%, respectively. To boost the color quality and efficiency, the hyper–OLEDs employing these two iridium complexes to sensitize the terminal emitter of *v*–DABNA are achieved in terms of the CE_max_, PE_max_ and *EQE*_max_ are 24.77 cd A^−1^, 19.71 lm W^−1^, and 29.50% for **Β–4–TMS** sensitized device, 26.91 cd A^−1^, 21.59 lm W^−1^, and 31.06% for **Β–5–TMS** sensitized device, respectively, corresponding to the CIE_y_ value down to 0.097, satisfying the National Institute of Standards and Technology (NIST) requirement for saturated blue in OLEDs display. With an intention to further improve the device stability, the fabricated saturated blue OLEDs to conduct the operational lifetime measurement. To note, such hyper–OLEDs depicted the converted lifetime (LT_50_) up to 4552 h at the luminance of 100 cd m^−2^ with the enhanced *EQE*_max_ up to 33.42% and FWHM as narrow as 17 nm, which has been the among the highest efficiency results reported for phosphor sensitized saturated–blue hyper–OLEDs.

## Conflict of Interest

The authors declare no conflict of interest.

## Supporting information



Supporting Information

## Data Availability

Research data are not shared.
