# Supplementary material for: New [3+2+1] Iridium Complexes as Effective Phosphorescent Sensitizers for Efficient Narrowband Saturated–Blue Hyper–OLEDs
Source: Adv Sci (Weinh). 2023 Aug 31;10(29):2301112. doi: 10.1002/advs.202301112 (PMC10582407; doi:10.1002/advs.202301112)
Supplement: Supplementary file 1 — Supporting Information [file ADVS-10-2301112-s001.pdf]

ADVANCED SCIENCE
Open Access

Supporting Information

for *Adv. Sci.*, DOI 10.1002/advs.202301112

New [3+2+1] Iridium Complexes as Effective Phosphorescent Sensitizers for Efficient Narrowband Saturated–Blue Hyper–OLEDs

Chengcheng Wu, Kai-Ning Tong, Kefei Shi, Zhaoyun Jin, Yuan Wu, Yingxiao Mu, Yanping Huo, Man-Chung Tang, Chen Yang, Hong Meng, Feiyu Kang and Guodan Wei**

Supporting Information

New [3+2+1] Iridium Complexes as Effective Phosphorescent Sensitizers for Efficient Narrowband Saturated−Blue Hyper−OLEDs

Chengcheng Wu[a,b], [#] Kai–Ning Tong[b], [#] Kefei Shi[a,b], Zhaoyun Jin[b], Yuan Wu[c], Yingxiao Mu[d], Yanping Huo[d], Man-Chung Tang[b], Chen Yang[c,*], Hong Meng[e], Feiyu Kang[a,b], Guodan Wei[a,b,*]

[a] Tsinghua−Berkeley Shenzhen Institute (TBSI), Tsinghua University, Shenzhen, 518055, China

[b] Institute of Materials Research, Tsinghua Shenzhen International Graduate School, Tsinghua University, Shenzhen, 518055, China

[c] PURI Materials, Inc, Shenzhen, 518133, China

[d] School of Chemical Engineering and Light Industry, Guangdong University of Technology, Guangzhou 510006, China

[e] School of Advanced Materials, Peking University, Shenzhen Graduate School, Peking University, Shenzhen 518055, China

# These two authors are contributed equally.

E−mail: david_yang@purimat.com; weiguodan@sz.tsinghua.edu.cn

Contents

Experimental section………………………………………………………...…….. S2–S7

NMR Spectra……………………………………………………..……..…...S8–S14

ESI–MS of iridium(III) complexes………………………..…...….................S15–S16

Summary of literature……………………………………………………….……S17

Thermal properties……………………………………………………….…….S18

Crystallography……………………………………………...………...…S19

Photophysical measurements……………………………………………….S20–S21

Electrochemical properties………………………………………………….S22–S23

Computational details and data…………………………….………………… S24–S32

Device……………………….……………………………………………...S33–S35

References…………………………………..…………………………S36–S37

EXPERIMENTAL SECTION

Materials Synthesis

Reagents and solvents employed were commercially available without further purification.

Characterization Method.

NMR spectra (^{1}H, ^{19}F NMR) were recorded on a 400 NMR spectrometer (Bruker). Chemical shifts in ^{1}H NMR spectra were referenced to tetramethylsilane (TMS) at 0.00 ppm. The absorption and phosphorescence spectra were performed using a UV−vis spectrophotometer (Cary 5000 UV–vis–NIR, Agilent, USA) and a spectrofluorometer (Edinburgh Instruments Ltd FS5), respectively. Electrochemical measurements were performed with a PalmSens4 electrochemical work station with platinum−carbon as working electrode, platinum wire as the counter electrode, and a saturated calomel electrode (SCE) in saturated KCl aqueous solution as the reference electrode. The cyclic voltammogram was referenced to the ferrocene/ferrocenium couple at a scan rate of 100 mV s^{-1}. Thermogravimetric analysis (TGA) was performed on a Mettler TGA2 thermogravimeter by measuring the weight loss from 25°C to 100°C at a rate of 10°C/min under nitrogen. After 15 minutes, heating from 100°C to 800°C at a rate of 10°C/min under nitrogen.

Device Fabrication.

The ITO (indium−tin oxide) coated glass substrates were first cleaned in deionized water, acetone, and ethanol, then dried in an oven and treated by ultraviolet ozone exposure for 20 min. Device fabrication were performed with a FS−450 (Suzhou Fangsheng) chamber. All organic layers were thermally evaporated at a rate of 0.5−1.5 Å s^{-1} at a pressure of *ca*. 7.5 $\times 10^{-7}$ Torr. A Liq layer (2.5 nm) was deposited at a rate of 0.2 Å s^{-1}. The Al cathode was deposited at a rate of 4 Å s^{-1}; the active area of the diode segments was 3×3 mm^2. The PH−OLEDs devices performance including EL spectra, current density−voltage−luminance (*J*–*V*–*L*) curves and Commission Internationale de L′Eclairage (CIE) coordinates were characterized by a Keithley 2400 semiconductor characterization system.

General Synthesis of main ligands.

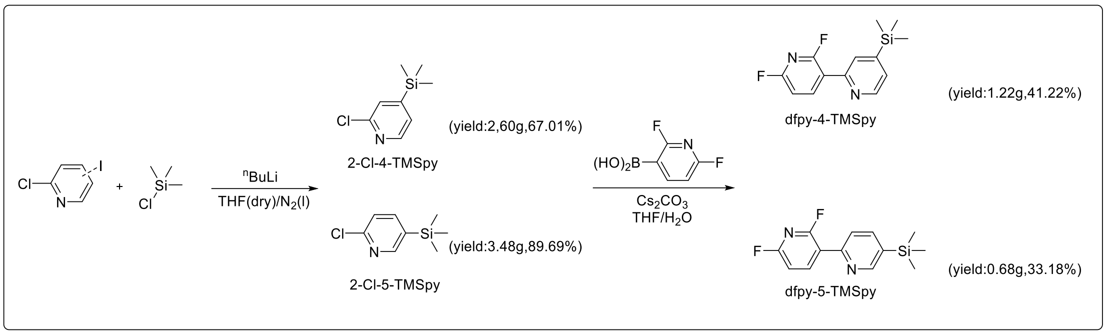

Scheme S1. Synthetic route for main ligands.

2–(2′,4′–Difluoropyridyl)– 4–trimethylsilylpyridine (dfpy–4–TMSpy):

2,4–Difluoropyridylboronic acid (2.65 g, 16.8 mmol), Cs_2CO_3 (18.25g, 56 mmol) and $Pd(PPh_3)_4$ (0.647 g, 0.56 mmol) were placed in a 250 mL two–necked round bottom flask equipped with a condenser. The reaction flask was evacuated and filled with N_2 gas three times. THF (70 mL), H_2O (30 mL) and 2–chloro–4–trimethylsilylpyridine (2.08 g, 11.20 mmol) were then added. The reaction mixture was heated to reflux for 24 h in an N_2 atmosphere and cooled to room temperature. THF was removed by evaporation and the residue was poured into CH_2Cl_2 (150 mL). The formed precipitate was removed by filtration through paper, and the filtrate was washed with 1 M NaOH (2 x 50 mL) and sat. NaCl (aq) (50 mL), dried over Na_2SO_4 and filtered. After evaporation of the solvent, the crude mixture was purified by column chromatography on silica gel (EtOAc / PE= 1:30) to provide 2–(2′,4′–Difluoropyridyl)–4–trimethylsilylpyridine (dfpy–4–TMSpy) (1.22 g, 41.22 %) as a transparent oil.

^{1}H NMR (400 MHz, Chloroform–d) δ 8.71 – 8.56 (m, 2H), 7.93 (dt, J = 2.3, 1.1 Hz, 1H), 7.41 (dd, J = 4.6, 1.0 Hz, 1H), 6.97 (ddd, J = 8.2, 3.0, 0.8 Hz, 1H), 0.34 (s, 9H). ^{19}F NMR (376 MHz, Chloroform–d) δ −68.35 (d, J = 10.3 Hz), −69.56 (d, J = 10.7 Hz).

2–(2′,4′–Difluoropyridyl)– 5–trimethylsilylpyridine (dfpy–5–TMSpy):

2,4–Difluoropyridylboronic acid (2.65 g, 16.8 mmol), Cs_2CO_3 (18.25g, 56 mmol) and $Pd(PPh_3)_4$ (0.647 g, 0.56 mmol) were placed in a 250 mL two–necked round bottom

flask equipped with a condenser. The reaction flask was evacuated and filled with N_2 gas three times. THF (70 mL), H_2O (30 mL) and 2−chloro−5−trimethylsilylpyridine (2.08 g, 11.20 mmol) were then added. The reaction mixture was heated to reflux for 24 h in an N_2 atmosphere and cooled to room temperature. THF was removed by evaporation and the residue was poured into CH_2Cl_2 (150 mL). The formed precipitate was removed by filtration through paper, and the filtrate was washed with 1 M NaOH (2 x 50 mL) and sat. NaCl (aq) (50 mL), dried over Na_2SO_4 and filtered. After evaporation of the solvent, the crude mixture was purified by column chromatography on silica gel (EtOAc / PE=1:30) to provide 2−(2′,4′−Difluoropyridyl)−5−trimethylsilylpyridine (dfpy−5−TMSpy) (0.68 g, 33.18 %) as a yellow oil.

^{1}H NMR (400 MHz, Chloroform−*d*) δ 8.78 (t, *J* = 1.4 Hz, 1H), 8.69 (dt, *J* = 9.6, 8.0 Hz, 1H), 7.90 (dd, *J* = 7.8, 1.9 Hz, 1H), 7.81 (ddd, *J* = 7.8, 2.4, 1.1 Hz, 1H), 6.97 (ddd, *J* = 8.3, 3.0, 0.9 Hz, 1H), 0.34 (s, 9H). ^{19}F NMR (376 MHz, Chloroform−*d*) δ −68.07 (d, *J* = 10.7 Hz), −69.25 (d, *J* = 10.4 Hz).

General Synthesis of Iridium Complexes.

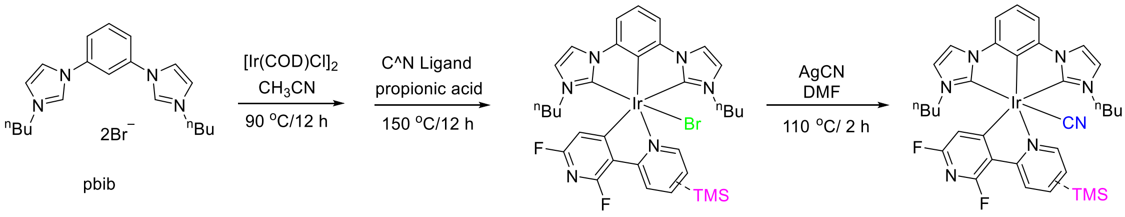

Scheme S2. Synthetic route for iridium complexes.

Under the protection of N_2, 1,3−bis(3′−butylimidazolyl−1′−yl)benzene dibromide **(pbib)** (0.90 mmol, 432.6 mg) and $[Ir(COD)Cl]_2$ (0.45 mmol, 300.0 mg) were added to a mixture of triethylamine (3 mL) and acetonitrile (45 mL) in Schlenk tube. The suspension was heated at 90 °C for 12 h. After cooling, the solvent was removed off by rotary evaporation to give yellow solid. Then the yellow solid was dissolved in propionic acid (45 mL), and 2−(2′,4′−difluoropyridyl)−4−trimethylsilylpyridine (0.90 mmol) and triethylamine (1 ml) was added to the solution and heated at 150°C under N_2. After cooling to room temperature, propionic acid was distilled under the low

pressure. The crude product was purified by silica gel column to afford the intermediate product. Then, the intermediate product was recrystallized in CH_2Cl_2/diethyl ether.

Under the protection of N_2, the intermediate product and the double molar quantity of AgCN were dissolved in a round–bottom flask containing 60 mL of *N*,*N*–dimethylformamide (DMF). The suspension was heated at 110 °C under N_2 for 2 h. After cooling, the solvent was filtered and removed off by rotary evaporation to afford the crude product. The crude product was purified by silica gel column to afford the expected product. Then, the expected product was recrystallized in CH_2Cl_2/diethyl ether.

dfpy–4–TMSpy–Br* (PB–4–TMS)*, yellow solid 369mg, total yield: 47.86 %

^{1}H NMR (400 MHz, Chloroform–*d*) δ 10.46 (d, *J* = 5.4 Hz, 1H), 8.49 (s, 1H), 7.51 (dd, *J* = 5.5, 1.2 Hz, 1H), 7.48 (d, *J* = 2.0 Hz, 2H), 7.24 (dd, *J* = 9.1, 6.4 Hz, 1H), 7.18 (d, *J* = 6.6 Hz, 2H), 6.77 (d, *J* = 2.0 Hz, 2H), 5.55 (d, *J* = 1.8 Hz, 1H), 3.29 (ddd, *J* = 13.3, 10.9, 5.6 Hz, 2H), 3.19 (ddd, *J* = 13.3, 10.6, 5.5 Hz, 2H), 1.46 – 1.37 (m, 2H), 1.07 – 1.00 (m, 2H), 0.87 – 0.78 (m, 2H), 0.74 – 0.66 (m, 8H), 0.45 (s, 9H). ^{19}F NMR (376 MHz, Chloroform–*d*) δ –70.13 (d, *J* = 10.2 Hz), –74.38 (d, *J* = 10.1 Hz). ESI–MS calcd for $C_{34}H_{42}F_2IrN_6Si$:$[M–Br]^+$, 777.2519, found 777.2521.

dfpy–5–TMSpy–Br* (PB–5–TMS)*, yellow solid 240mg, total yield: 31.13 %

^{1}H NMR (400 MHz, Chloroform–*d*) δ 10.63 (q, *J* = 0.9 Hz, 1H), 8.34 (d, *J* = 8.1 Hz, 1H), 8.07 – 7.96 (m, 1H), 7.48 (d, *J* = 2.1 Hz, 2H), 7.26 – 7.21 (m, 1H), 7.20 – 7.16 (m, 2H), 6.77 (d, *J* = 2.1 Hz, 2H), 5.52 (d, *J* = 1.8 Hz, 1H), 3.35 – 3.14 (m, 4H), 1.37 (dq, *J* = 9.5, 4.4 Hz, 2H), 1.24 – 1.14 (m, 2H), 0.91 (dd, *J* = 14.3, 8.3 Hz, 2H), 0.82 (t, *J* = 5.2 Hz, 2H), 0.73 (t, *J* = 6.8 Hz, 6H), 0.45 (s, 9H).^{19}F NMR (376 MHz, Chloroform–*d*) δ –69.81 (d, *J* = 10.0 Hz), –74.20 (d, *J* = 10.2 Hz). ESI–MS calcd for $C_{34}H_{42}F_2IrN_6Si$:$[M–Br]^+$, 777.2519, found

Under the protection of N_2, the intermediate product and the double molar quantity of AgCN were dissolved in a round–bottom flask containing 60 mL of *N*,*N*–dimethylformamide (DMF). The suspension was heated at 110 °C under N_2 for 2

h. After cooling, the solvent was filtered and removed off by rotary evaporation to afford the crude product. The crude product was purified by silica gel column to afford the expected product. Then, the expected product was recrystallized in CH_2Cl_2/diethyl ether.

dfpy–4–TMSpy–CN* (B–4–TMS)*, white solid 195 mg, total yield: 84.6%

^{1}H NMR (400 MHz, Chloroform–*d*) δ 10.22 (d, *J* = 5.5 Hz, 1H), 8.51 (s, 1H), 7.51 (d, *J* = 2.1 Hz, 2H), 7.46 (dd, *J* = 5.4, 1.2 Hz, 1H), 7.27 (d, *J* = 7.0 Hz, 1H), 7.19 – 7.15 (m, 2H), 6.82 (d, *J* = 2.1 Hz, 2H), 5.52 (s, 1H), 3.34 – 3.15 (m, 4H), 1.45 (dd, *J* = 11.4, 6.1 Hz, 2H), 1.05 (dt, *J* = 11.0, 5.7 Hz, 2H), 0.86 (dt, *J* = 11.8, 5.8 Hz, 2H), 0.74 – 0.68 (m, 8H), 0.44 (s, 9H). ^{19}F NMR (376 MHz, Chloroform–*d*) δ −69.63 (d, *J* = 9.6 Hz), −74.35 (d, *J* = 9.6 Hz).

dfpy–5–TMSpy–CN* (B–5–TMS)*, yellow solid 207mg, total yield: 89.8 %

^{1}H NMR (400 MHz, Chloroform–*d*) δ 10.35 (dd, *J* = 1.7, 0.8 Hz, 1H), 8.36 (dt, *J* = 8.3, 1.0 Hz, 1H), 8.06 (dd, *J* = 8.1, 1.7 Hz, 1H), 7.51 (d, *J* = 2.1 Hz, 2H), 7.28 – 7.25 (m, 1H), 7.22 – 7.14 (m, 2H), 6.82 (d, *J* = 2.1 Hz, 2H), 5.48 (t, *J* = 2.3 Hz, 1H), 3.35 – 3.16 (m, 4H), 1.47 – 1.37 (m, 2H), 1.20 (dddd, *J* = 13.4, 9.5, 5.2, 2.4 Hz, 2H), 1.03 – 0.90 (m, 2H), 0.89 – 0.78 (m, 2H), 0.75 (t, *J* = 7.1 Hz, 6H), 0.47 (s, 9H). ^{19}F NMR (376 MHz, Chloroform–*d*) δ −69.31 (d, *J* = 9.6 Hz), −74.13 (d, *J* = 9.6 Hz).

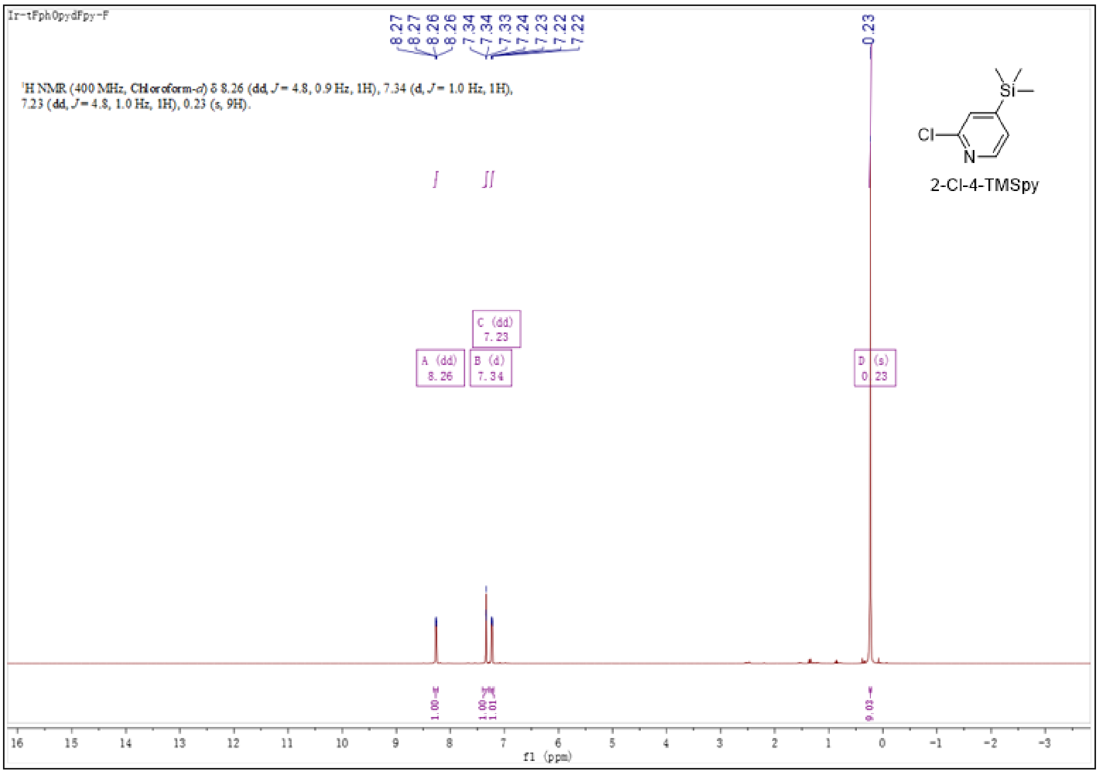

Figure S1. ^{1}H NMR (400MHz, $CDCl_3$) of ligand ***2–Cl–4–TMSpy***

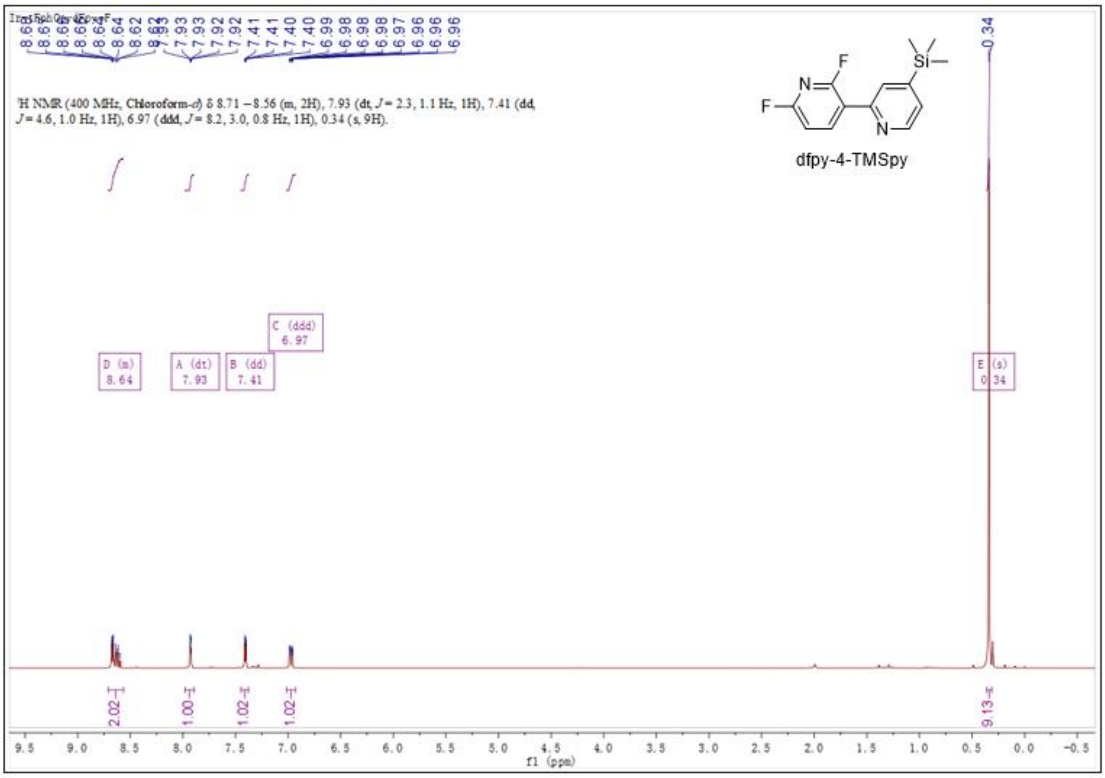

Figure S2. ^{1}H NMR (400MHz, $CDCl_3$) of ligand ***2–(2′,4′–Difluoropyridyl)–4–trimethylsilylpyridine (dfpy–4–TMSpy)***

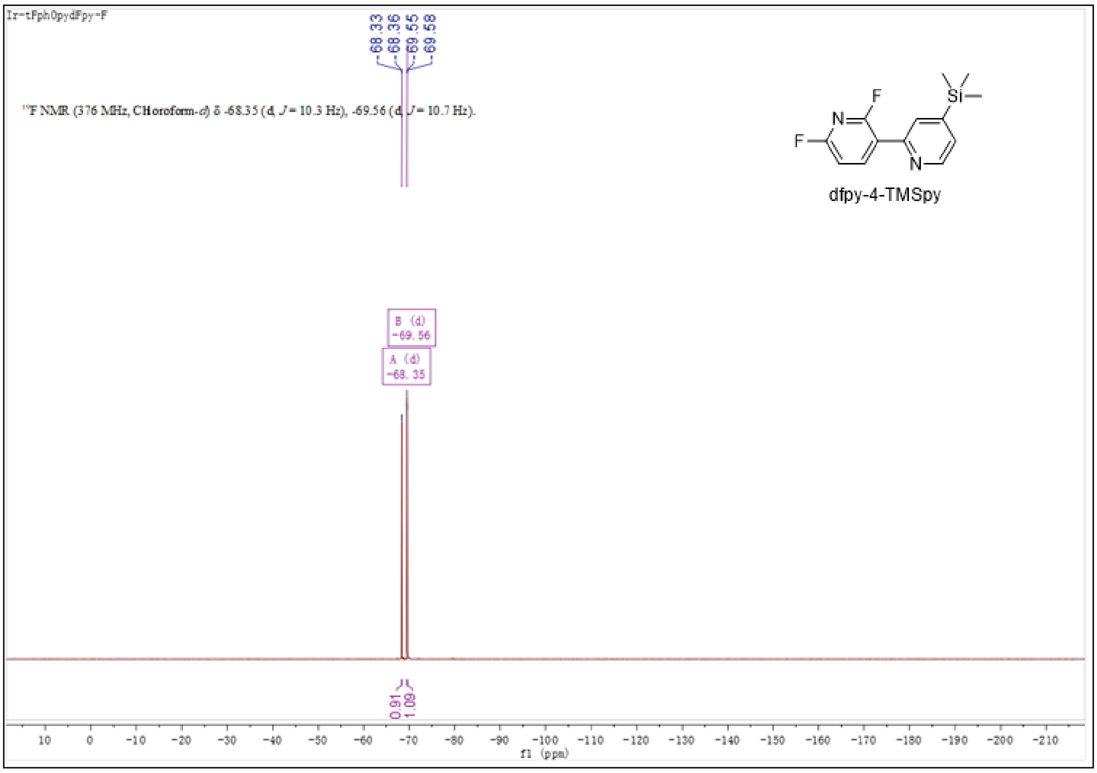

Figure S3. ^{19}F NMR (375MHz, $CDCl_3$) of ligand ***2–(2′,4′–Difluoropyridyl)–4–trimethylsilylpyridine (dfpy–4–TMSpy)***

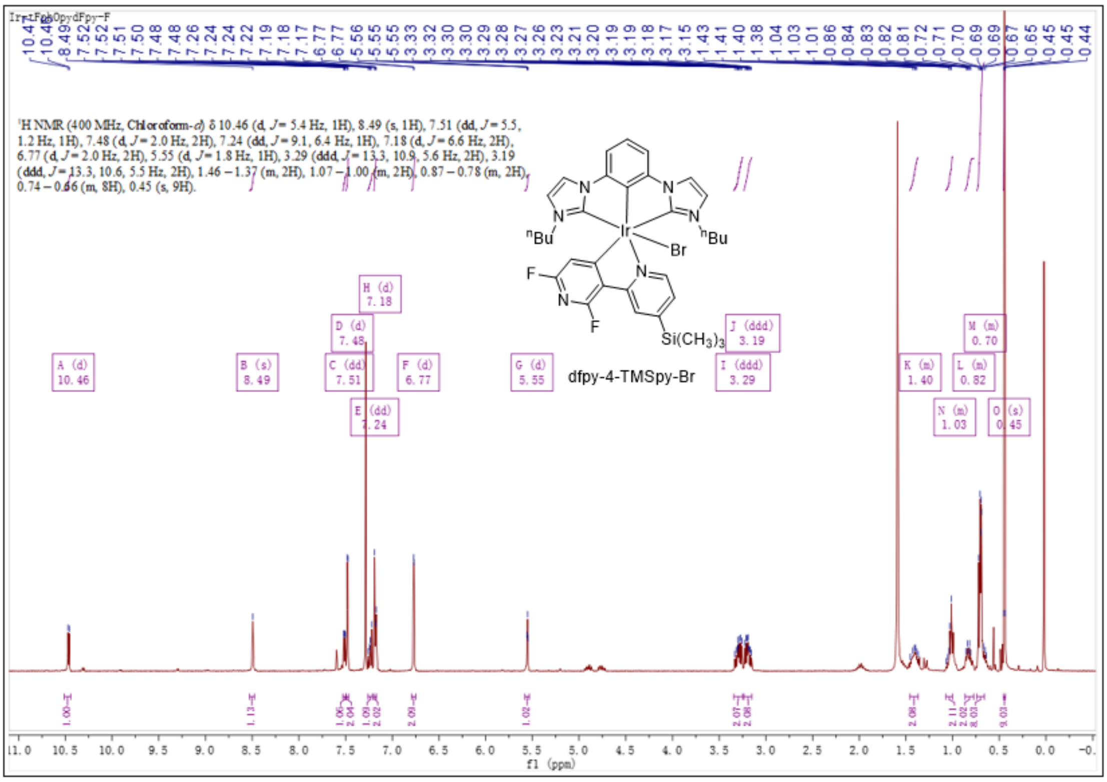

Figure S4. ^{1}H NMR (400MHz, $CDCl_3$) of iridium complex ***dfpy–4–TMSpy–Br*** **(PB–4–TMS)**

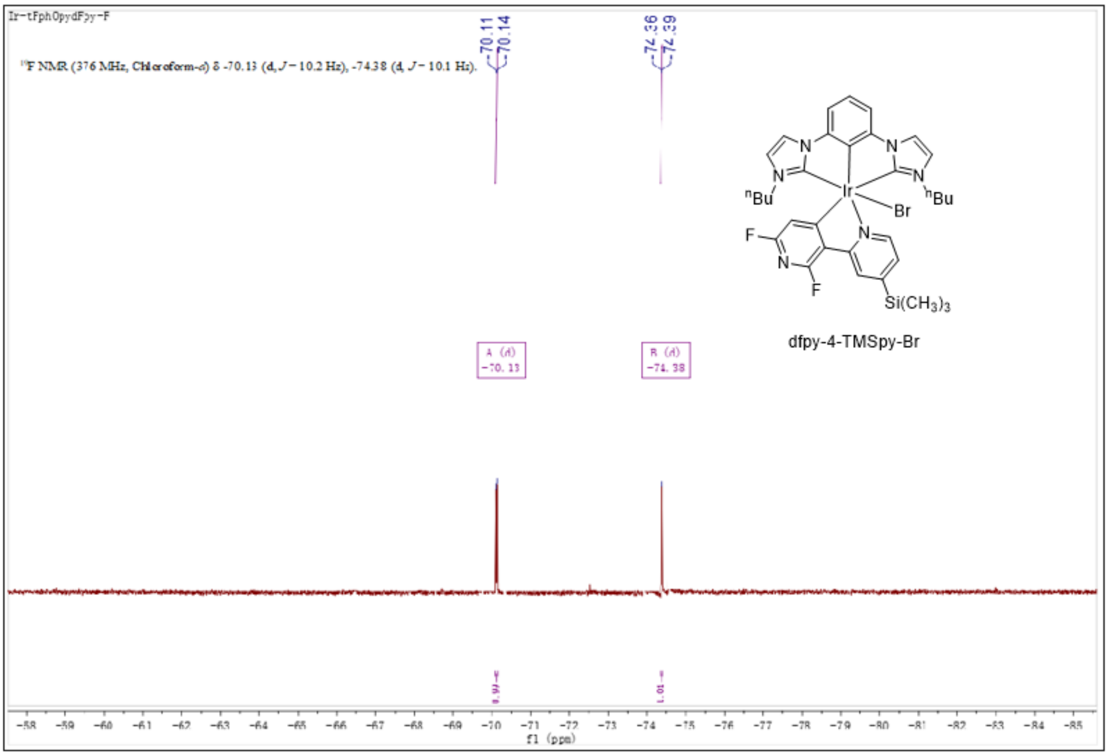

Figure S5. ^{19}F NMR (375MHz, $CDCl_3$) of iridium complex ***dfpy–4–TMSpy–Br*** **(PB–4–TMS)**

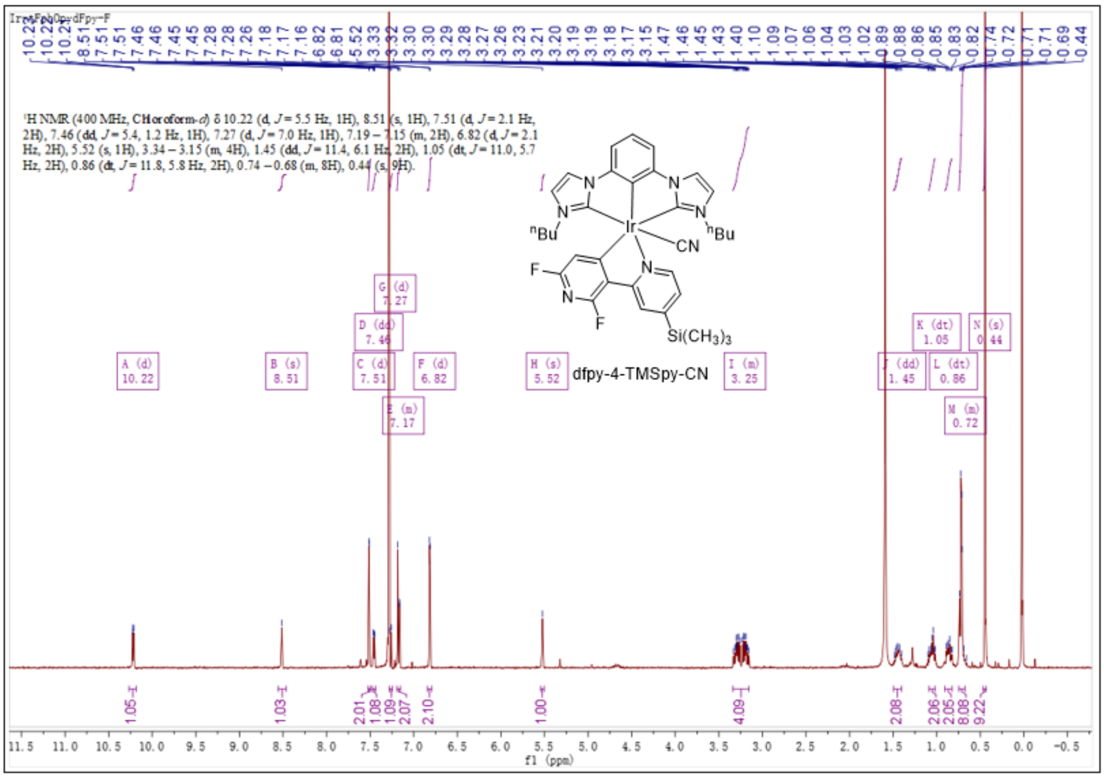

Figure S6. ^{1}H NMR (400MHz, $CDCl_3$) of iridium complex ***dfpy–4–TMSpy–CN*** **(PB–4–TMS)**

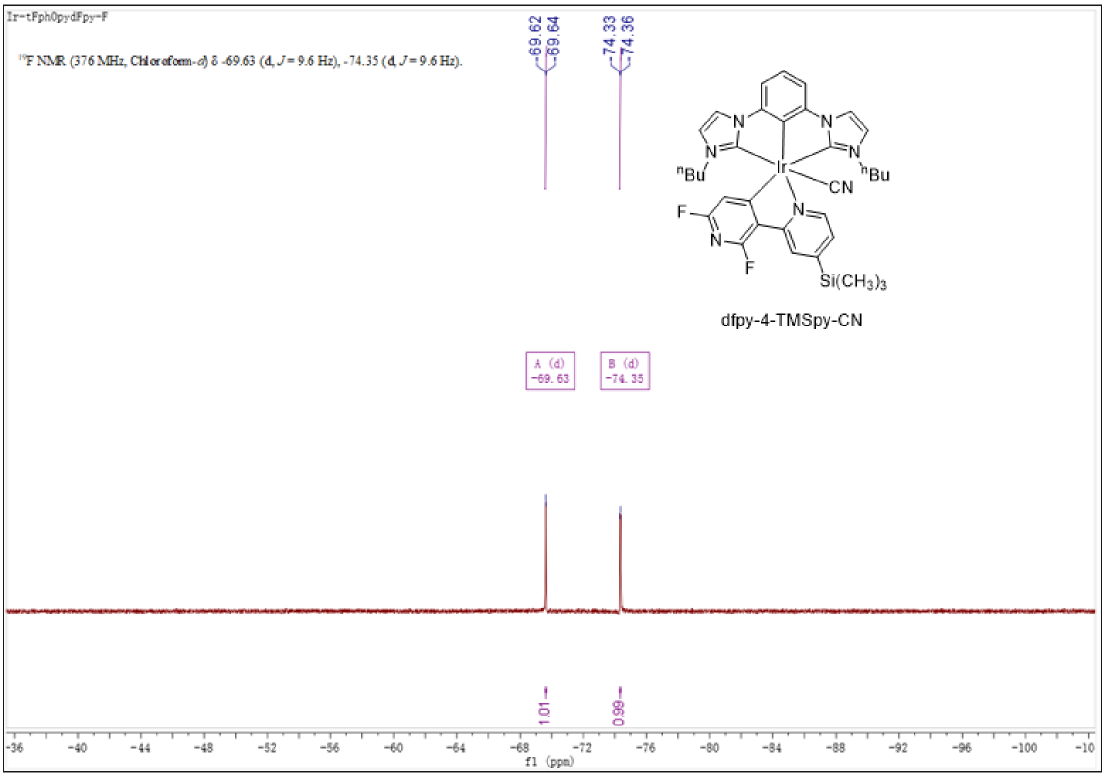

Figure S7. ^{19}F NMR (375MHz, $CDCl_3$) of iridium complex ***dfpy–4–TMSpy–CN* (B–4–TMS)**

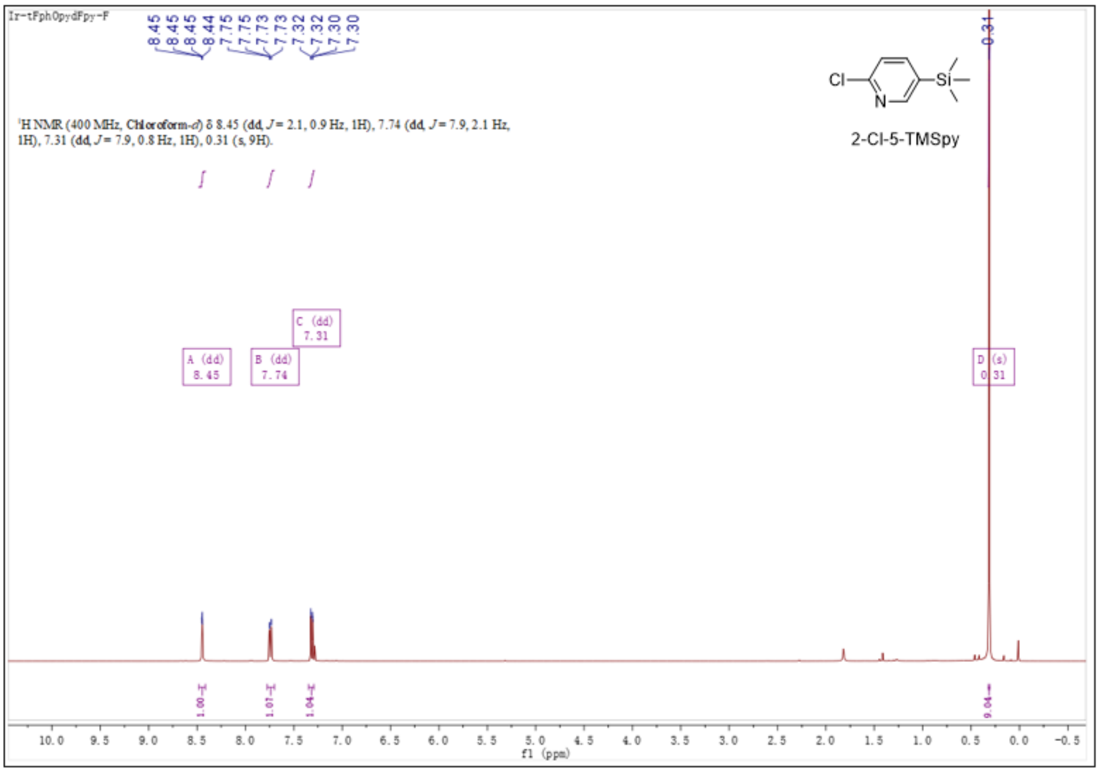

Figure S8. ^{1}H NMR (375MHz, $CDCl_3$) of ligand ***2–Cl–5–TMSpy)***

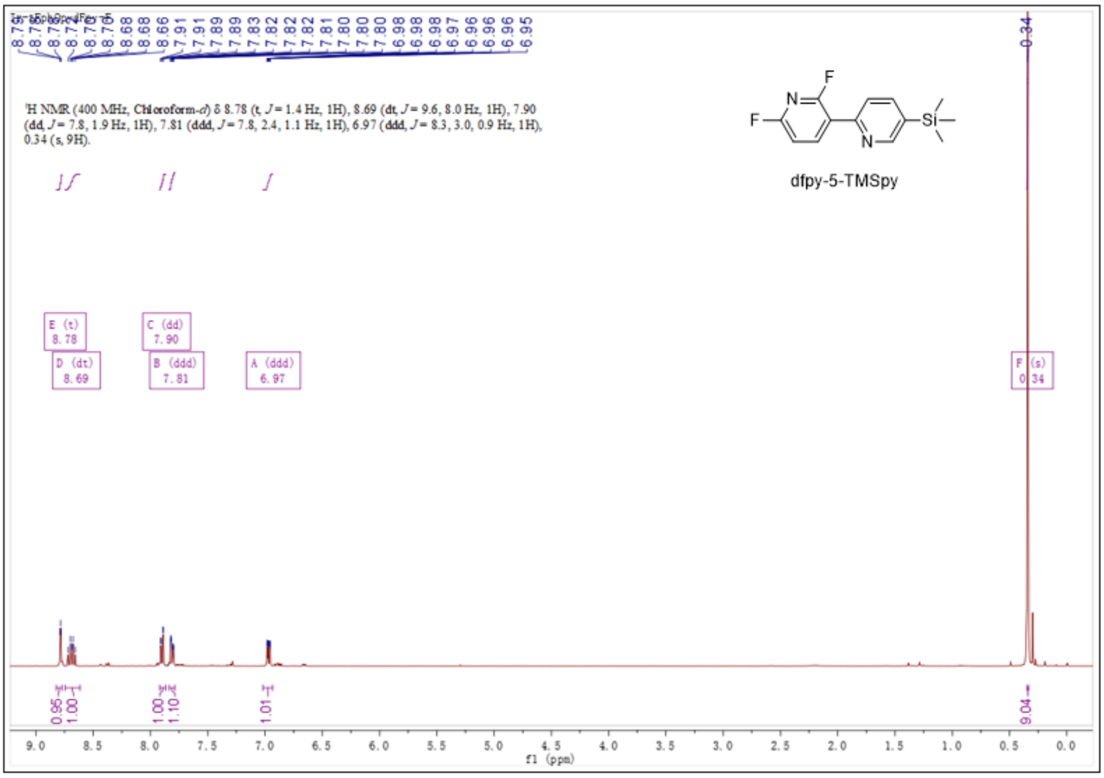

Figure S9. ¹H NMR (400MHz, $CDCl_3$) of ligand ***2–(2′,4′–Difluoropyridyl)–5–trimethylsilylpyridine (dfpy–5–TMSpy)***

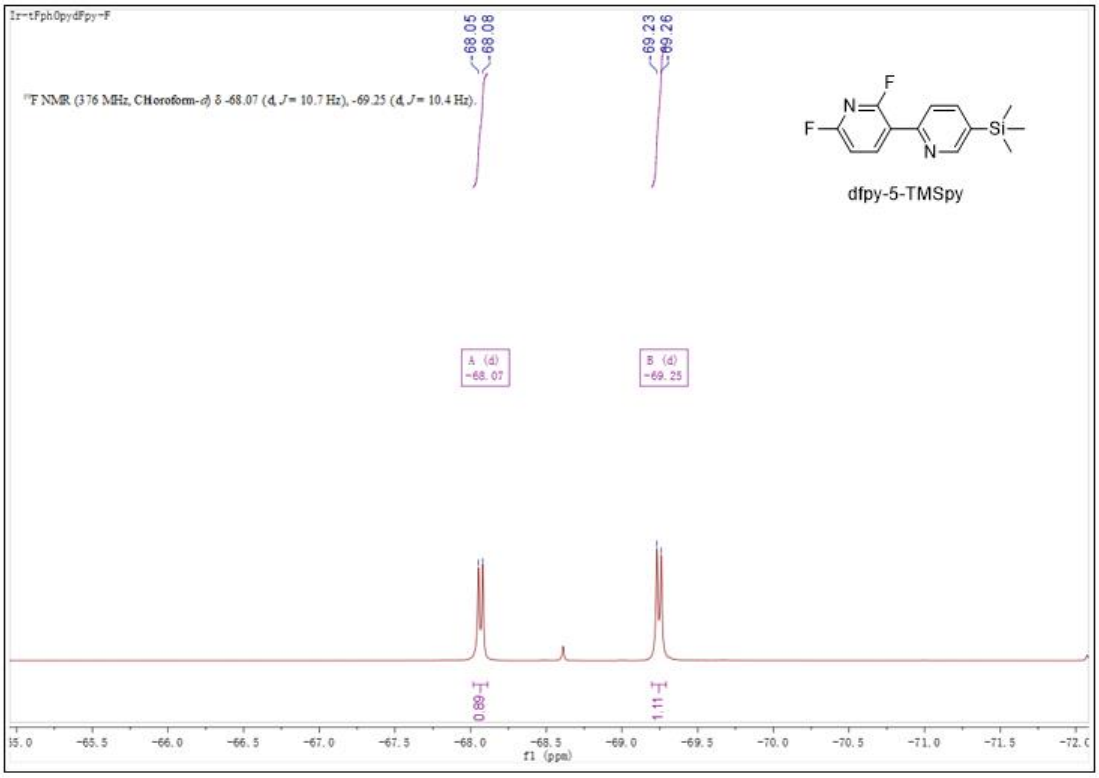

Figure S10. ¹⁹F NMR (375MHz, $CDCl_3$) of ligand ***2–(2′,4′–Difluoropyridyl)–5–trimethylsilylpyridine (dfpy–5–TMSpy)***

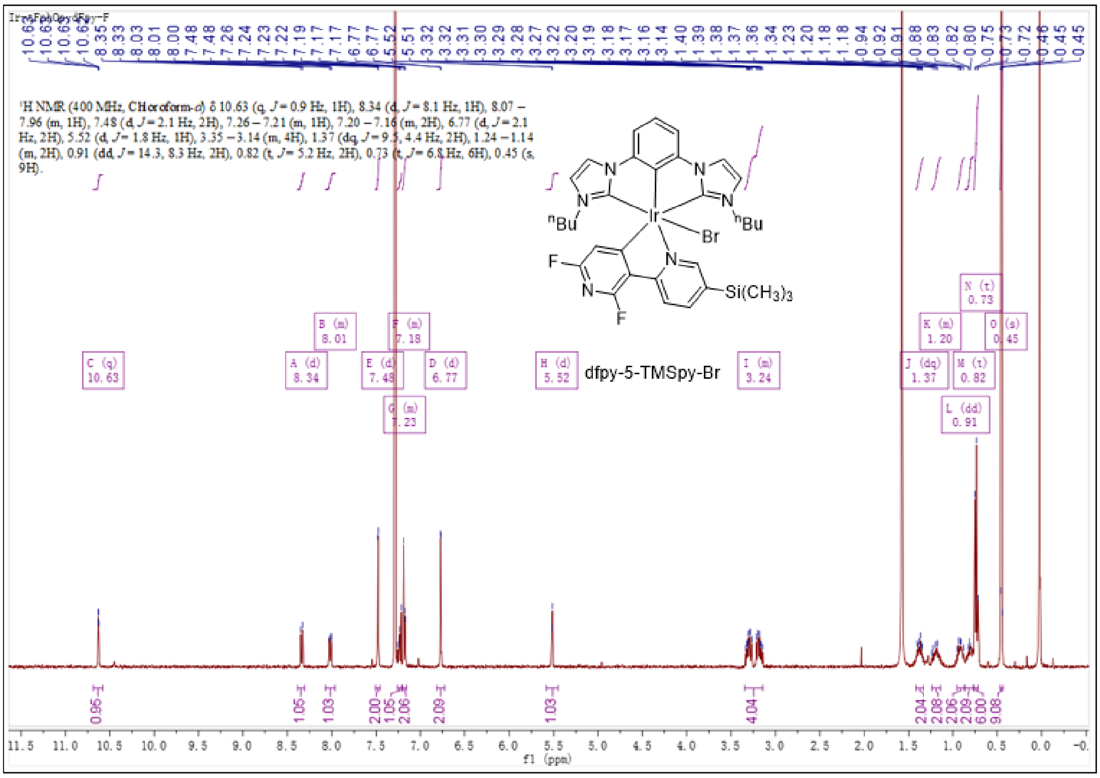

Figure S11. ^{1}H NMR (400MHz, $CDCl_3$) of iridium complex ***dfpy–5–TMSpy–Br* (PB–5–TMS)**

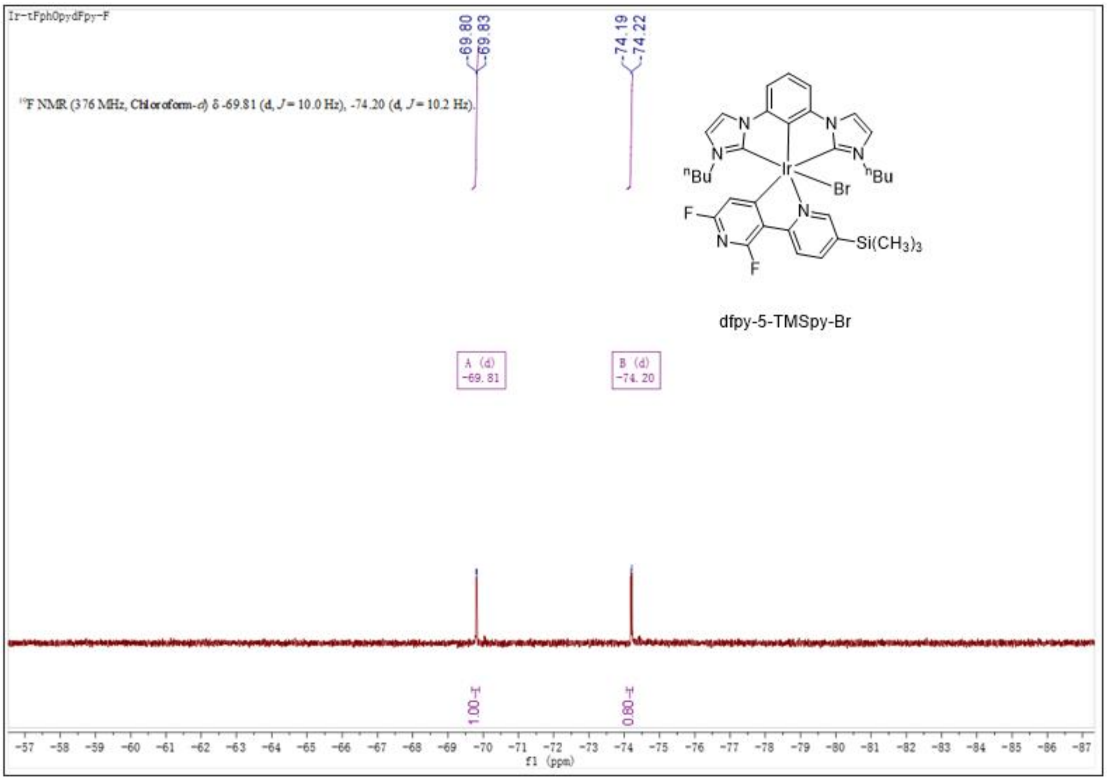

Figure S12. ^{19}F NMR (375MHz, $CDCl_3$) of iridium complex ***dfpy–5–TMSpy–Br* (PB–5–TMS)**

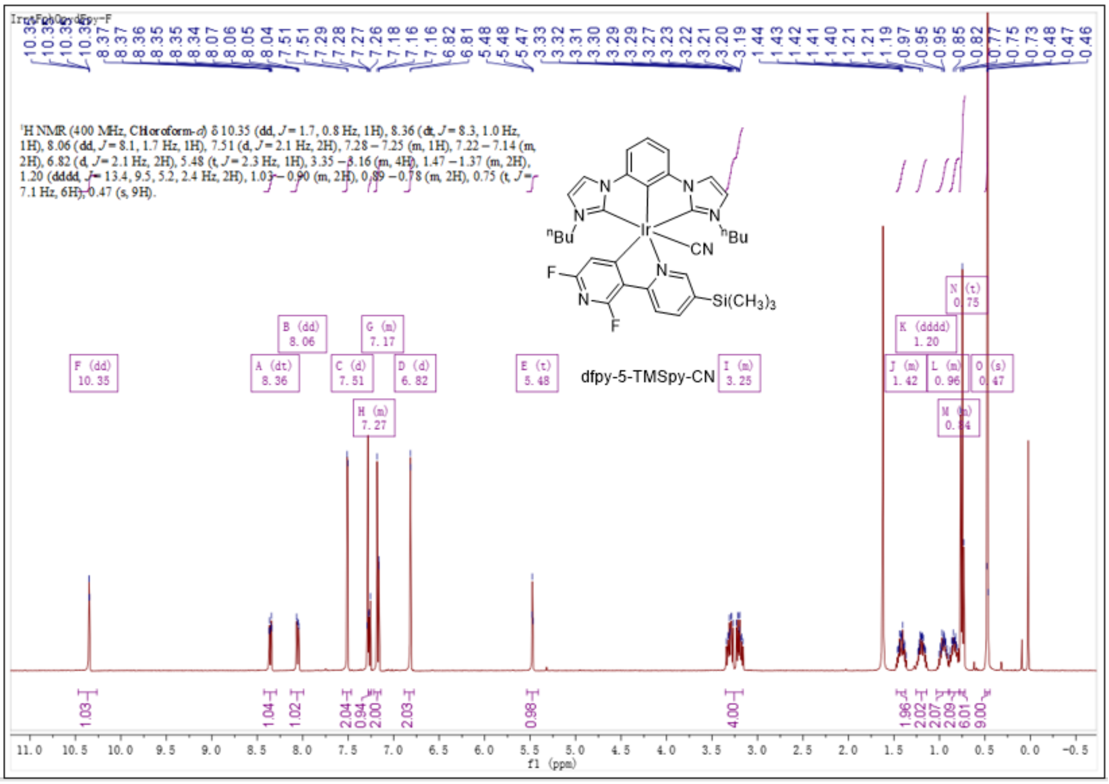

Figure S13. ^{1}H NMR (400MHz, $CDCl_3$) of iridium complex ***dfpy–5–TMSpy–CN*** **(B–5–TMS)**

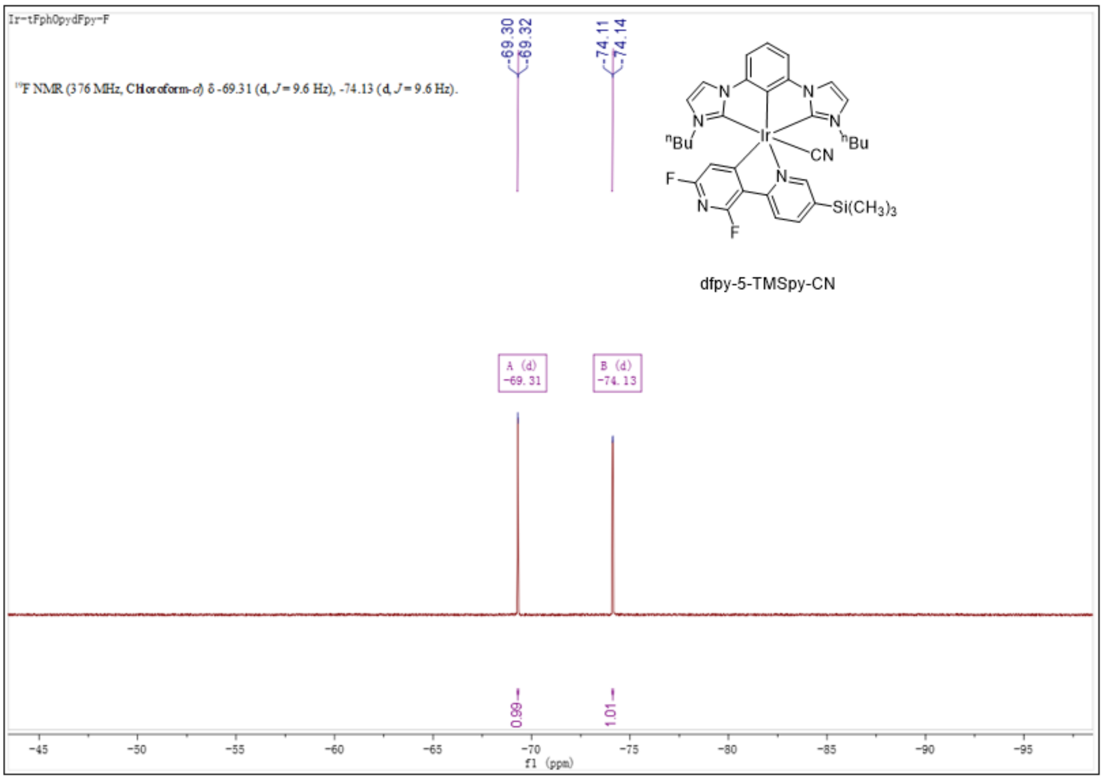

Figure S14. ^{19}F NMR (375MHz, $CDCl_3$) of iridium complex ***dfpy–5–TMSpy–CN*** **(B–5–TMS)**

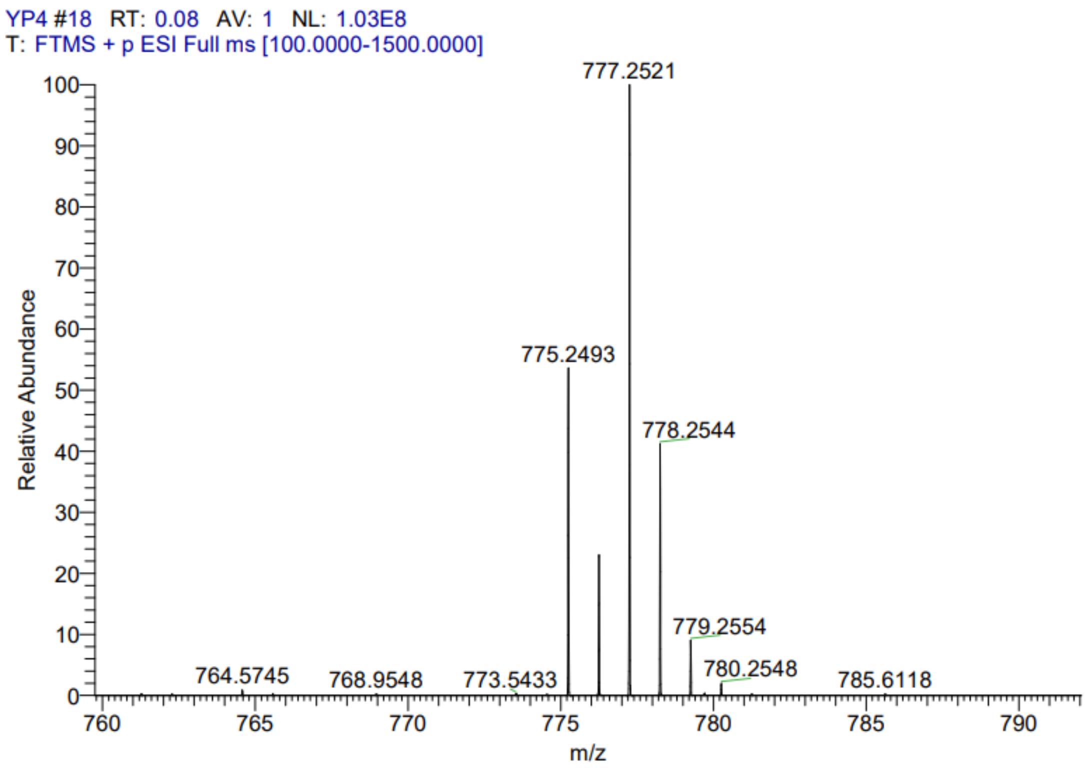

Figure S15. ESI–MS of iridium complex ***dfpy–4–TMSpy–Br*** **(PB–4–TMS)**

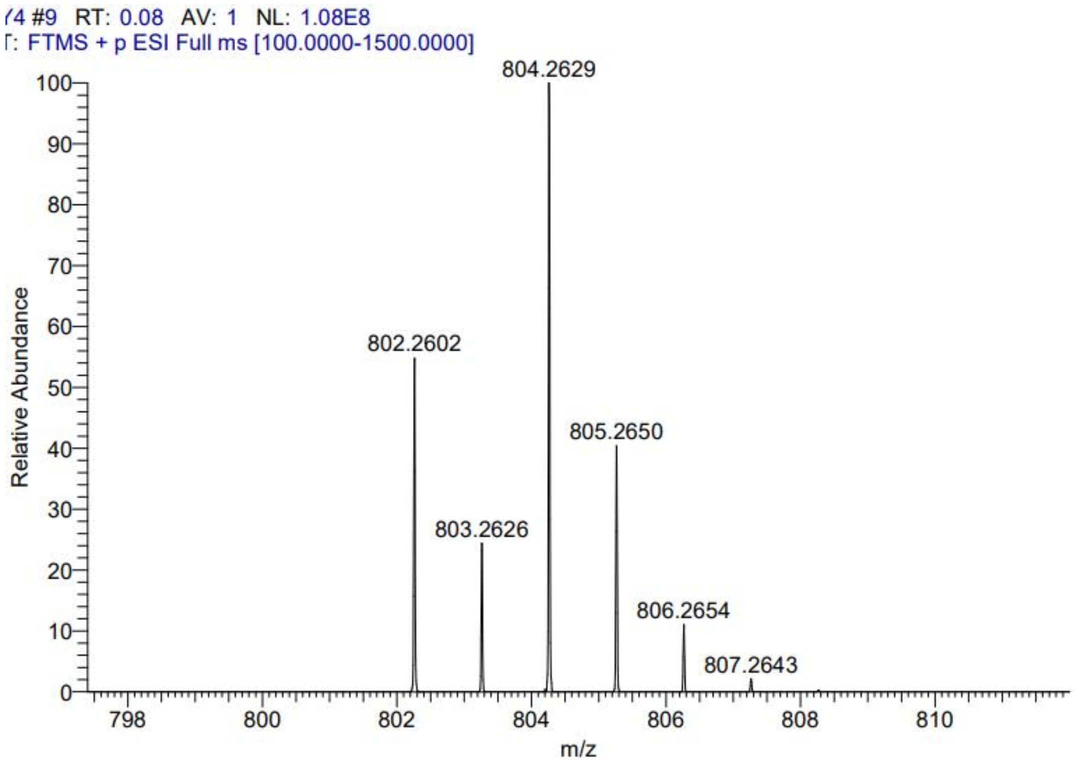

Figure S16. ESI–MS of iridium complex ***dfpy–4–TMSpy–CN*** **(B–4–TMS)**

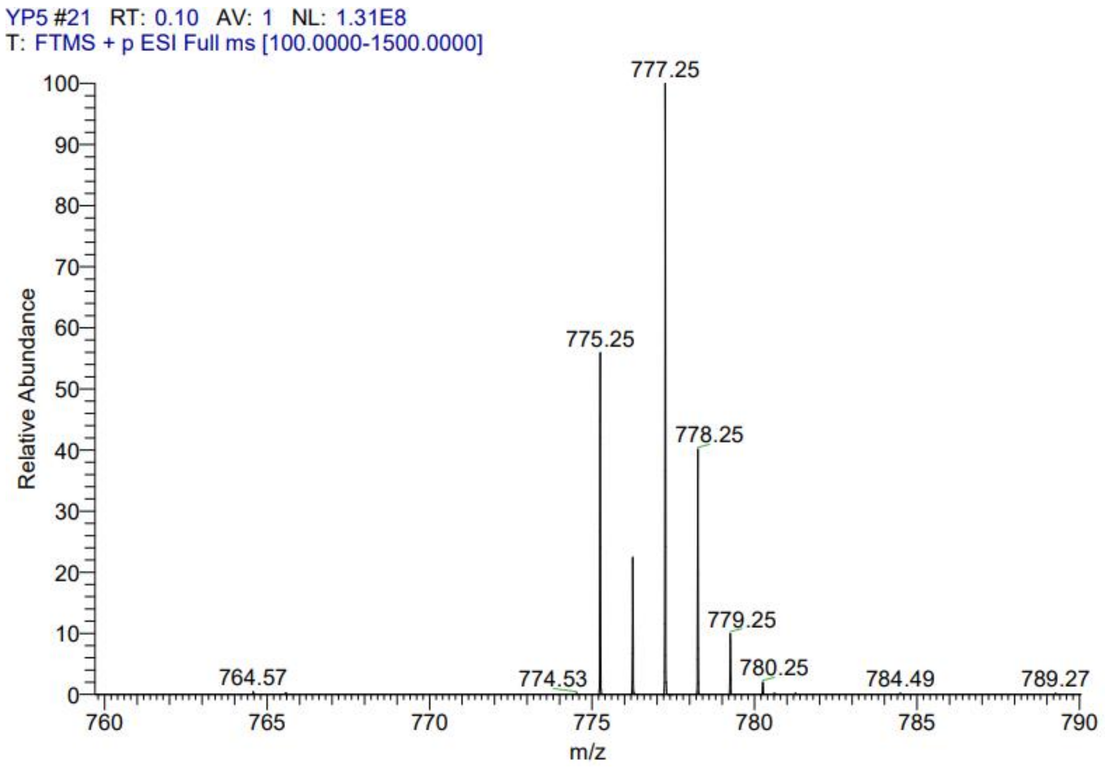

Figure S17. ESI−MS of iridium complex ***dfpy−5−TMSpy−Br*** **(PB−5−TMS)**

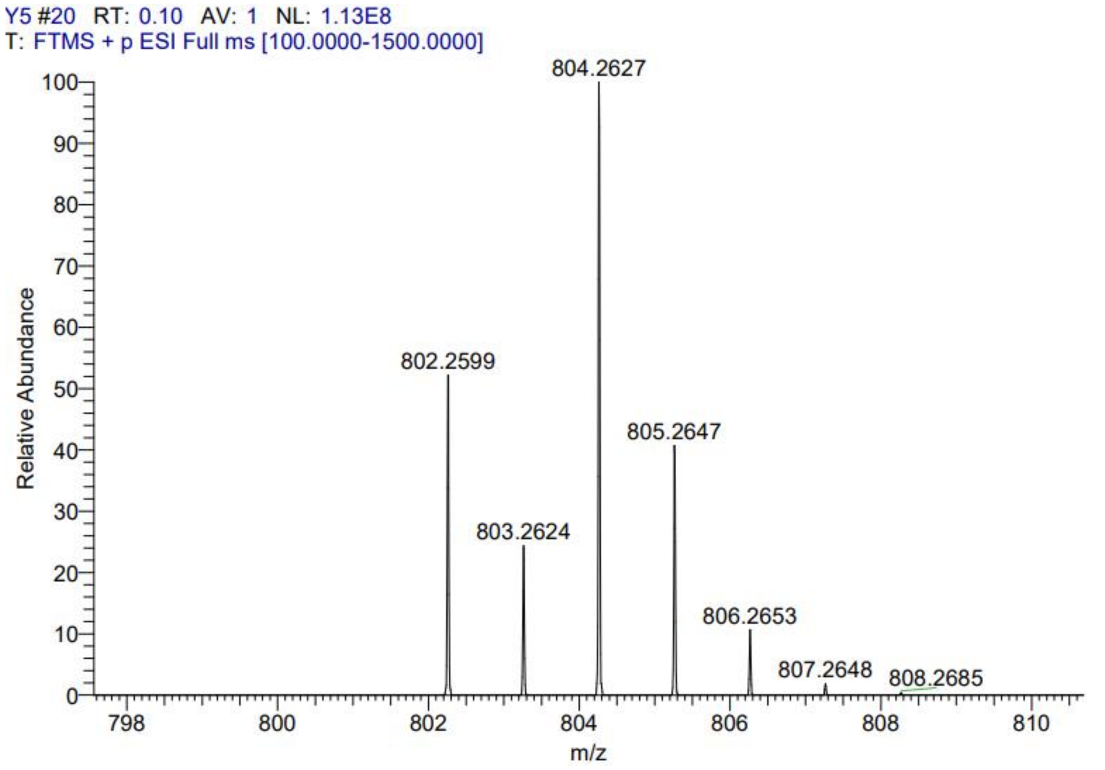

Figure S18. ESI−MS of iridium complex ***dfpy−5−TMSpy−CN*** **(B−5−TMS)**

Summary of literatures

Table S1. The reported BN−based blue−emitting (CIE_y < 0.20) hyper−OLEDs

Dopants	EQE $_{max/1000}$	$CIE_{(x,y)}$	FWMF(nm)	Operational lifetime (LT)	Ref.
**B–5–TMS** / *v*–DABNA[b]	31.06 / 22.74	(0.128, 0.097)	18	LT_{50} at 100 cd m^{-2}=3h	TW[a]
**B–5–TMS** / *v*–DABNA[c]	33.42 / 24.09	(0.119, 0.123)	17	LT_{50} at 100 cd m^{-2}=4552h	TW[a]
m–2–tBu / *v*–DABNA	22.0 / 14.9	(0.120, 0.155)	21	–	[1]
**6 /** *v*–DABNA	15.76/10.50	(0.13, 0.19)	20	–	[2]
**5** / *v*–DABNA	23.4 / 22.4	(0.13, 0.12)	18	LT_{50} at 1000 cd m^{-2}=259h	[3]
HDT1 / *v*–DABNA(tendem)	41 / 32	(0.13, 0.16)	19	LT_{95} at 1000 cd m^{-2}=18h	[4]
PtON7–dtb / *v*–DABNA	32.2 / 25.4	(0.111, 0.141)	17	LT_{50} at 1000 cd m^{-2}=156.3h	[5]
PPCzTrz / *v*–DABNA	33.0 / 25.2	(0.13, 0.20)	19	LT_{50} at 1000 cd m^{-2}=151h	[6]
CN–Ir / *v*–DABNA	27.3 / 23.3	(0.132, 0.162)	20	LT_{50} at 1000 cd m^{-2}=121h	[7]
f–tpb1 / *t*–DABNA	29.6 /–	(0.13, 0.11)	30	–	[8]
f–CF_3 / *t*–DABNA	23.8 / 10.4	(0.13, 0.14)	30	–	[9]
p4TCzPhBN / *t*–DABNA	32.5 / 23.2	(0.13, 0.12)	29	LT_{80} at 1000 cd m^{-2}>60h	[10]
DMAC–DPS / *t*–DABNA	31.4 / 19.8	(0.13, 0.15)	28	LT_{50} at 100 cd m^{-2}=32h	[11]
$Ir(cb)_3$ / *t*–DABNA	20.2 / 14.2	(0.129, 0.108)	27	LT_{50} at 1000 cd m^{-2}=16.2h	[12]
TDBA–SAF / pBP–DABNA–Me	30.1 / –	(0.133, 0.109)	22	–	[13]
3Cz2BN / **BN3**	37.6 / 26.2	(0.14, 0.08)	23	–	[14]
DtBuAc–DBT / **α–3BNMes**	15 / –	(0.15, 0.10)	49	–	[15]
DBA–BFICz / *v*–DABNA	38.8 / 23.6	(0.12, 0.15)	19	–	[16]
PtON-TBBI / *t*–DABNA	25.8	CIEy = 0.165	21	LT_{95} at 1000 cd m^{-2}=72.9h	[17]
DBA-DTMCz / *v*–DABNA	43.9 / 37.5	(0.12, 0.16)	21	–	[18]
B3 / *v*–DABNA	26.17 / 17.92	(0.116, 0.114)	2862 cm^{-1}	–	[19]

[a] TW refers to This Work;

[b] The terminal emitter, sensitizer and the host materials in emissive layer as followed: *v*–DABNA, **B–5–TMS** and CzSi;

[c] The terminal emitter, sensitizer and the host materials in emissive layer as followed: *v*–DABNA, **B–5–TMS** and SiCzCz:SiTrzCz2.

Thermal properties

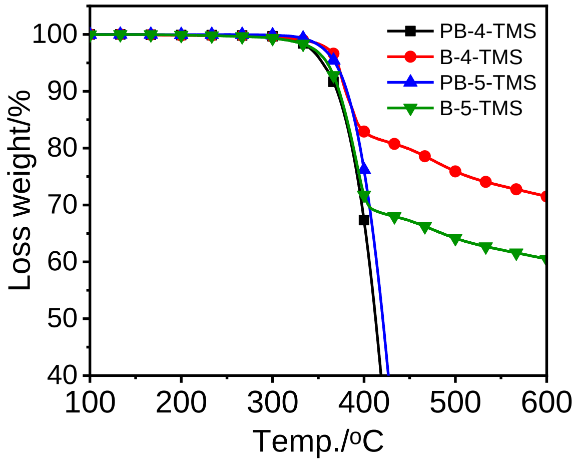

Figure S19. The thermogravimetric analysis (TGA) curves for the iridium complexes.

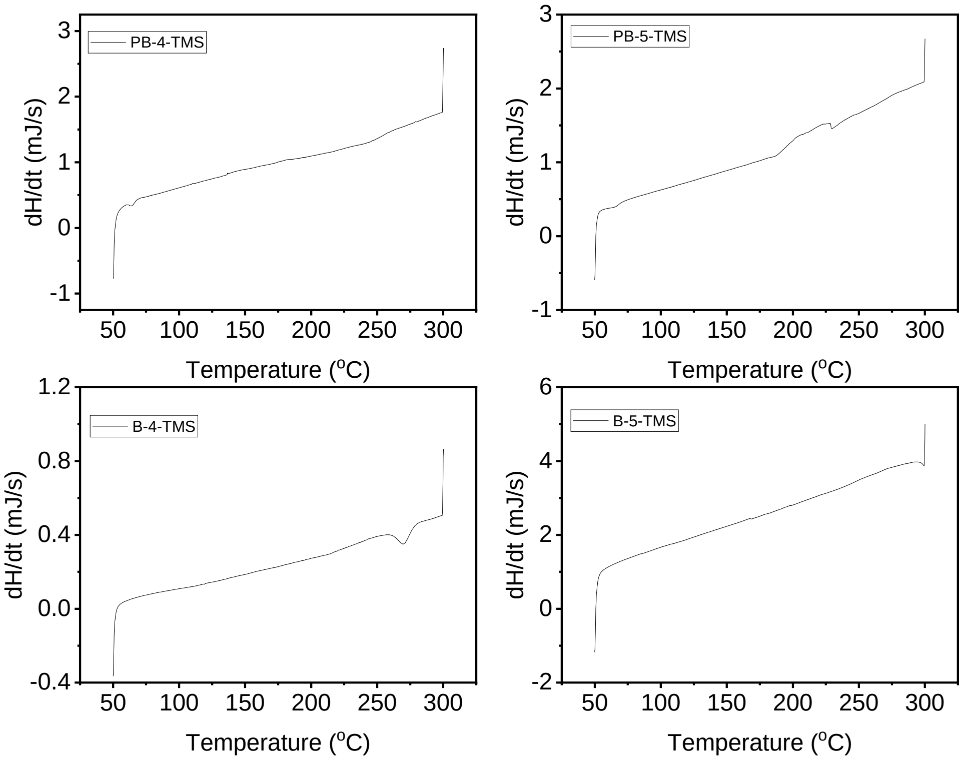

Figure S20. DSC analysis for the iridium complexes

Table S2. Thermal stabilities of the iridium complexes.

Complex	T_d^a / °C	T_g^b / °C
**PB–4–TMS**	371.3	136.3
**PB–5–TMS**	364.2	–
**B–4–TMS**	350.0	–
**B–5–TMS**	340.2	169.1

[a] Decomposition temperature (T_d) is defined as the temperature at which the complex shows a 5 % weight loss;[b] T_g is defined as glass transition temperature (T_g)

Crystallography

Table S3. Selected Bond Lengths and Angles for Complexes **PB–5–TMS** and **B–5–TMS**.

**Complex**	**PB–5–TMS**	**B–5–TMS**
Selected Bond Lengths(Å)		
Ir1–C1	2.057(4)	2.054(5)
Ir1–C4	1.969(3)	1.966(4)
Ir1–C10	2.054(3)	2.046(4)
Ir1–C21	1.978(3)	2.030(4)
Ir1–N5	2.144(3)	2.1267(19)
Ir1–X[a]	2.6039(4)	2.039(4)
Selected Bond Angles(deg)		
C10–Ir1–N5	102.69(12)	102.59(15)
C10–Ir1–C1	155.61(14)	155.40(18)
C1–Ir1–N5	101.65(12)	101.82(14)
C21–Ir1–N5	79.18(13)	78.45(15)
C21–Ir1–C10	92.20(13)	91.07(17)
C21–Ir1–C1	90.96(13)	90.84(17)
C4–Ir1–N5	173.59(12)	172.44(15)
C4–Ir1–C10	77.90(14)	77.69(19)
C4–Ir1–C1	77.75(14)	77.71(18)
C4–Ir1–C21	94.43(14)	94.00(18)
N5–Ir1–X[a]	94.02(8)	93.45(14)
C10–Ir1–X[a]	90.76(9)	91.58(17)
C1–Ir1–X[a]	88.94(10)	89.96(17)
C21–Ir1–X[a]	173.04(10)	171.85(18)
C4–Ir1–X[a]	92.35(10)	94.09(18)

[a] X represent the atoms which are coordinated to the iridium center of the monodentate ligand (X = Br for **PB–5–TMS**; X = C34 for **B–5–TMS**).

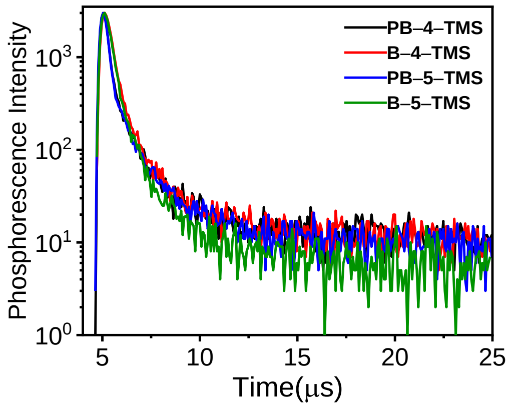

Figure S21. Time-resolved photoluminescence decay profiles of the iridium complexes in degassed CH_2Cl_2 at 298 K.

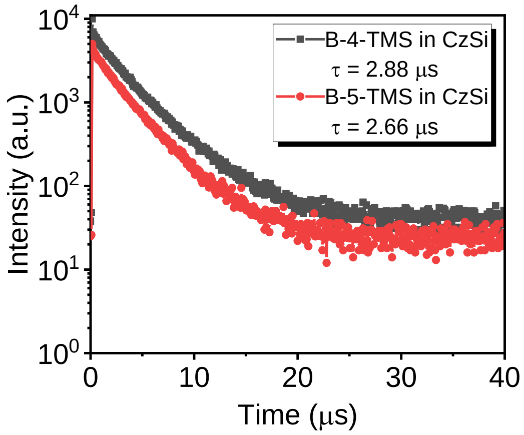

Figure S22. Time-resolved photoluminescence decay profiles of the iridium complexes in thin film at 298 K.

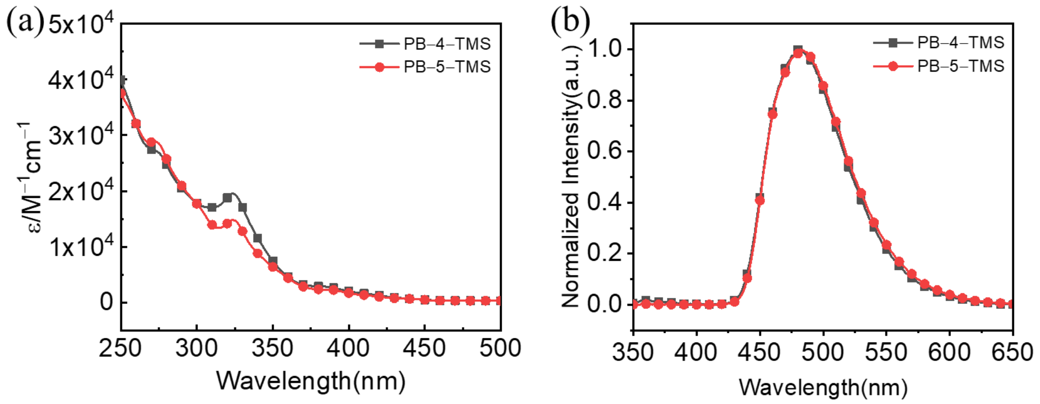

Figure S23. (a) Absorption (b) Emission spectra in dichloromethane ($c = 2 \times 10^{-5}$ M) at 298 K;

Table S4. Solution state photophysical properties of iridium (III) complexes.

Complex	Absorption[a] λ_{max}(nm)	Emission at 298 K in CH_2Cl_2 Solution				
	ε x10^4 M^{-1} cm^{-1}	λ_{max}[b](nm)	Φ_{PL}[c]	τ (μs)	K_r[d](10^6 s^{-1})	K_{nr}[e]($10^6 s^{-1}$)
**PB–4–TMS**	276(2.62),328(1.97),387(0.45)	485	18	0.30	0.60	2.73
**PB–5–TMS**	276(3.09),328(1.61),391(0.39)	488	14	0.29	0.48	2.97

[a] Absorption spectrum was measured in dichloromethane solution; [M] = 2.0×10^{-5}.

[b] Emission spectrum was measured in degassed dichloromethane; [M] = 2.0×10^{-5}, λ_{exc} = 320 nm.

[c] Phosphorescence quantum efficiency measured by absolute method using integrating sphere.

[d,e] Radiative as well as non–radiative rate constants were deduced by the Φ_{PL} of solution state and τ_{obs} according to two equations: $k_r = \Phi_{PL} / \tau_{obs}$, $k_{nr} = (1-\Phi_{PL}) / \tau_{obs}$.

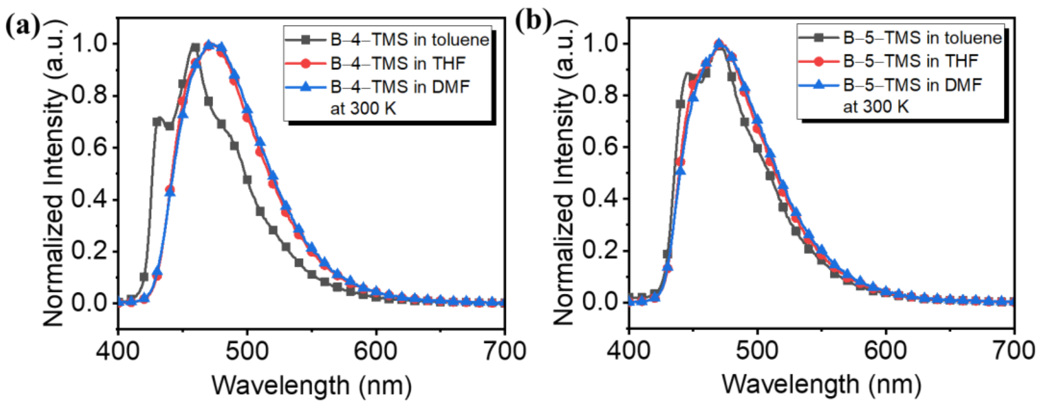

Figure. S24 Normalized solvent–dependent emission spectra of (a) B–4–TMS and (b) B–5–TMS in various solvents at 300 K.

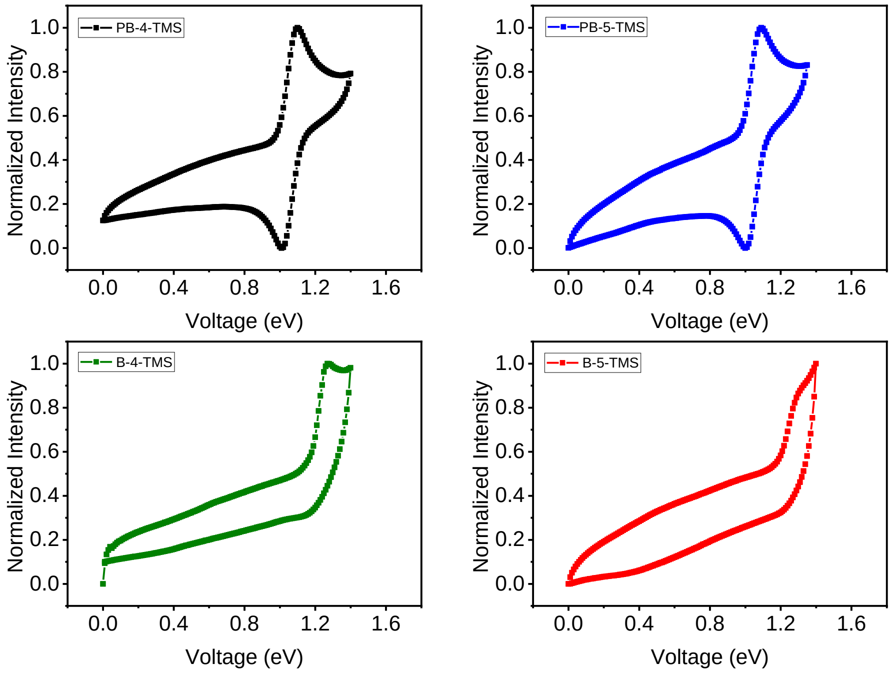

Figure S25. Cyclic voltammograms of the iridium complexes in degassed CH_2Cl_2 for oxidation

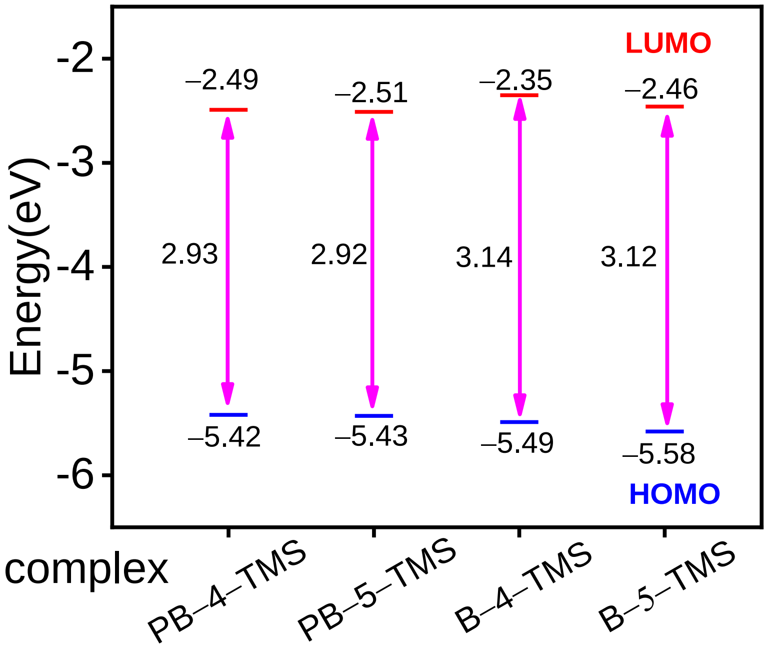

Figure S26. The estimated HOMO and LUMO energy levels of the iridium complexes.

Table S5. Electrochemical data by CV experiments for PB−4−TMS and PB−5−TMS.

Complex	Experiment			
	$E_{1/2}^{OX}$ (V)[a]	E_g (eV)[b]	HOMO (eV)[c]	LUMO (eV)[d]
PB−4−TMS	1.06	2.93	−5.42	−2.49
PB−5−TMS	1.04	2.92	−5.43	−2.51

[a] The oxidation potential data were record in CH_2Cl_2 with 0.1 M $^{n}Bu_4NPF_6$;

[b]Energy gap is achieved from the onset of the absorption, where $E_g = 1240 / \lambda_{absorp.\ onset}$.

[c]LUMO = HOMO + E_g

[d]E_{HOMO} levels were estimated from electrochemical potentials, i.e. $E_{HOMO} = -e(E_{pa}+(4.8-F_c^+/F_c))$ or $E_{HOMO} = -e(E_{1/2}^{Ox}+(4.8-F_c^+/F_c))$. ($F_c^+/F_c$ = 0.46 V in CH_2Cl_2).

Computational details and data

Density functional theory (DFT) and time–dependent density functional theory (TDDFT) calculations were performed to understand the geometries and the electronic structures of **B–4–TMS** and **B–5–TMS** using the Gaussian 16 package.[20] The vibrational frequency calculations at the same level were carried out to verify that every optimized structure is an energy minimum (no imaginary frequency). PBE0[21], 6–31G*(IANI2DZ)[22–23] was used for both the geometry optimization and TDDFT calculations. The solvent effects were examined using the self–consistent reaction field (SCRF) method based on PCM models.[24–25] The choice of solvents (DCM, a dielectric constant ε = 8.93) was based on the solvent media for experiments. Calculated first 15 singlet excited state energies (λ / nm), the associated oscillator strengths (*f*) and the nature of the transitions at the optimized ground state (S0) geometries of **B–4–TMS** and **B–5–TMS** in the dichloromethane (DCM) by TD–PBE0. The values in the parentheses are the % contributions of that particular configuration state function (CSF).

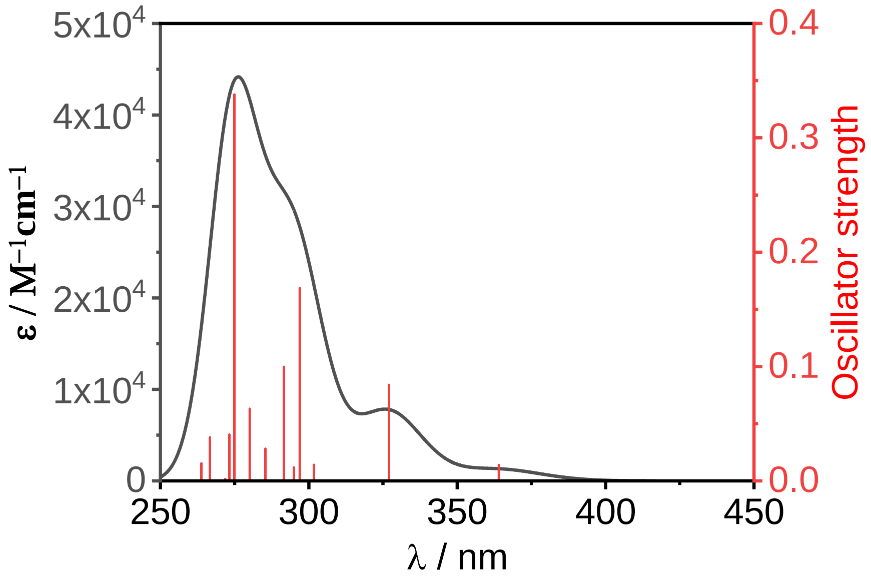

Figure S27. Simulated absorption spectrum of **B–4–TMS**. The red vertical lines refer to the unbroadened oscillator strengths of the singlet–singlet transitions, and the black line is the fitting line of the UV–vis absorption.

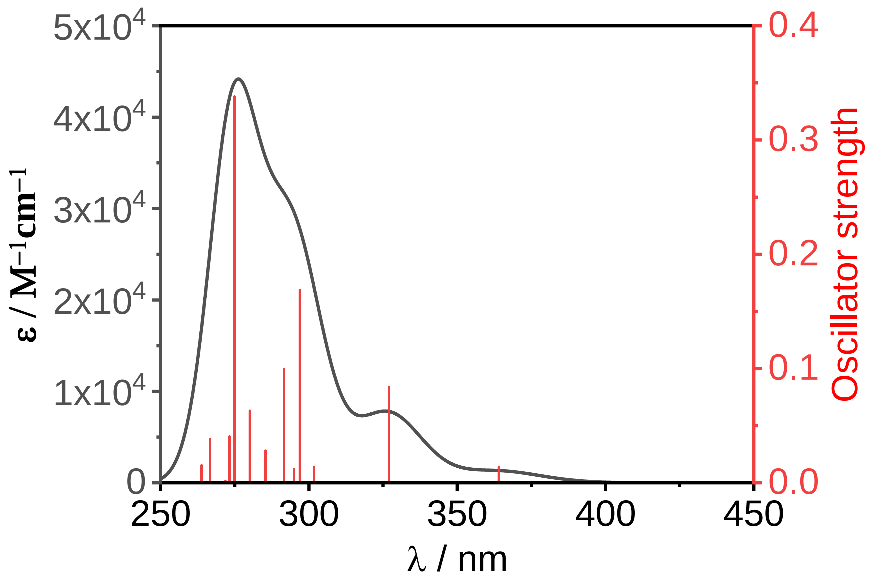

Figure S28. Simulated absorption spectrum of **B–5–TMS**. The red vertical lines refer to the unbroadened oscillator strengths of the singlet–singlet transitions, and the black line is the fitting line of the UV−vis absorption.

Table S6. Calculated first 15 singlet excited state energies (λ / nm), the associated oscillator strengths (*f*) and the nature of the transitions at the optimized ground state (S_0) geometries of **B–4–TMS** and **B–5–TMS** in the dichloromethane (DCM) by TD–PBE0. The values in the parentheses are the % contributions of that particular configuration state function (CSF).

**B–4–TMS**	Energy (eV)	λ (nm)	*f*	Major contribs
1	3.2885	377.02	0.0001	HOMO(H)→LUMO(L) (98.30%)
2	3.406	364.02	0.0139	H-1→L(96.10%)
3	3.7917	326.99	0.084	H-2→L(92.90%)
4	4.0088	309.28	0.0003	H→L+1(98.10%)
5	4.1094	301.71	0.0141	H-1→L+1(81.00%)<br>H-3→L (11.10%)
6	4.1757	296.92	0.1688	H-3→L (66.70%)<br>H-1→L+1 (15.90%)<br>H-2→L+1 (10.80%)
7	4.2035	294.95	0.0118	H-4→L (96.00%)
8	4.2516	291.62	0.0998	H→L+2 (93.50%)
9	4.3446	285.38	0.0281	H-5→L (87.80%)<br>H-2→L+1 (5.70%)
10	4.4268	280.08	0.0632	H-1→L+2 (67.70%)<br>H→L+4 (19.20%)<br>H→L+3 (5.30%)
11	4.51	274.91	0.3379	H-2→L+1 (61.00%)<br>H-3→L (10.60%)<br>H→L+4 (7.70%)<br>H→L+3 (6.50%)
12	4.5376	273.24	0.0406	H-1→L+4 (66.30%)<br>H-1→L+3 (14.50%)<br>H→L+3 (10.10%)

13	4.56	271.9	0.0015	H→L+3 (72.40%)<br>H-1→L+4 (11.60%)<br>H→L+4 (10.00%)
14	4.6489	266.7	0.0381	H→L+4 (49.80%)<br>H-1→L+2 (17.80%)<br>H-2→L+2 (7.10%)<br>H-2→L+1 (6.60%)
15	4.6993	263.84	0.0154	H-2→L+2 (77.10%)<br>H-1→L+3 (6.30%)
**B–5–TMS**	Energy (eV)	λ (nm)	*f*	Major contribs
1	3.2885	377.02	0.0001	HOMO(H)→LUMO(L) (98.30%)
2	3.406	364.2	0.014	H-1→L(96.10%)
3	3.797	326.9	0.084	H-2→L(92.90%)
4	4.0089	309.27	0.0003	H→L+1(98.10%)
5	4.1095	301.7	0.0415	H-1→L+1(80.90%)<br>H-3→L (11.10%)
6	4.1757	296.92	0.1688	H-3→L (66.70%)<br>H-1→L+1 (16.0%)<br>H-2→L+1 (10.80%)
7	4.2035	294.95	0.0118	H-4→L (96.00%)
8	4.2516	291.62	0.0998	H→L+2 (93.50%)
9	4.3446	285.38	0.0281	H-5→L (87.80%)<br>H-2→L+1 (5.70%)
10	4.4268	280.08	0.0632	H-1→L+2 (67.70%)<br>H→L+4 (19.20%)<br>H→L+3 (5.30%)
11	4.5101	274.9	0.3381	H-2→L+1 (61.00%)<br>H-3→L (10.60%)<br>H→L+4 (7.80%)<br>H→L+3 (10.20%)
12	4.5377	273.23	0.0405	H-1→L+4 (66.40%)<br>H-1→L+3 (14.40%)<br>H→L+3 (10.20%)
13	4.56	271.9	0.0015	H→L+3 (72.50%)<br>H-1→L+4 (11.60%)<br>H→L+4 (9.90%)
14	4.648	266.7	0.0381	H→L+4 (49.80%)<br>H-1→L+2 (17.80%)<br>H-2→L+2 (7.10%)<br>H-2→L+1 (6.60%)
15	4.6993	263.84	0.0154	H-2→L+2 (77.10%)<br>H-1→L+3 (6.40%)

Figure S29. Spatial plots (isovalue = 0.02) of selected molecular orbitals of **B–4–TMS** at the optimized PBE0 ground–state geometry.

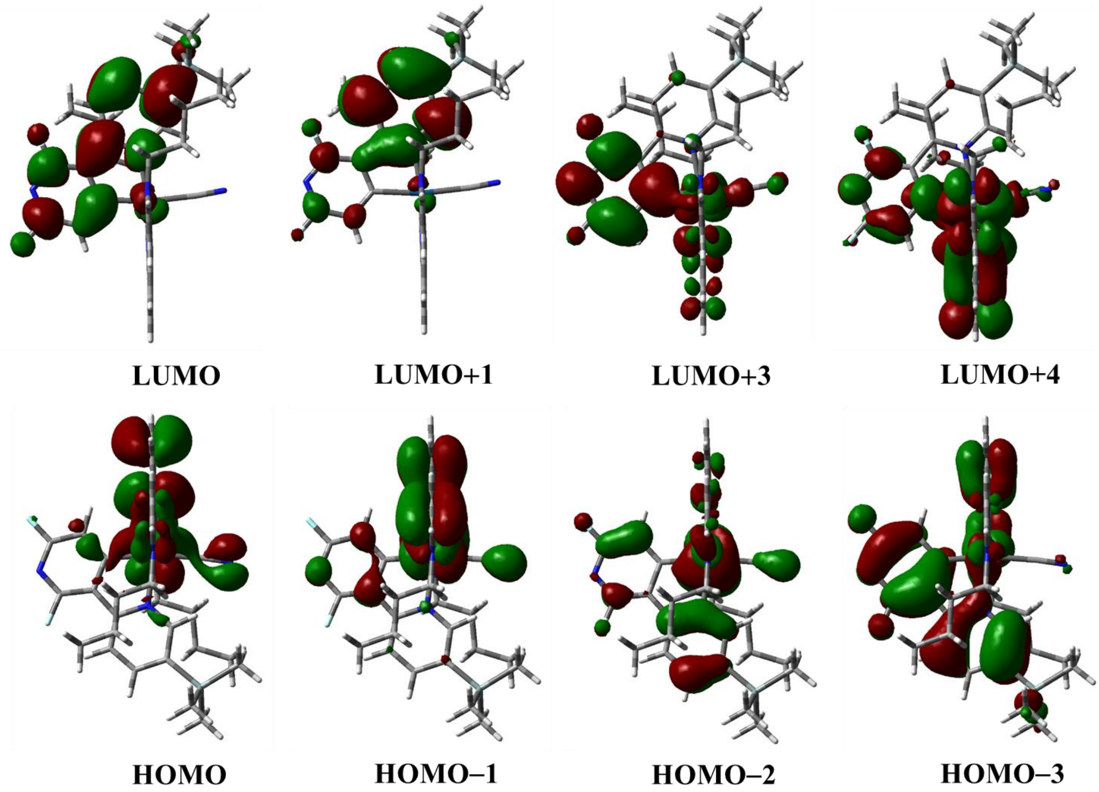

Figure S30. Spatial plots (isovalue = 0.03) of selected molecular orbitals of **B–5–TMS** at the optimized PBE0 ground–state geometry.

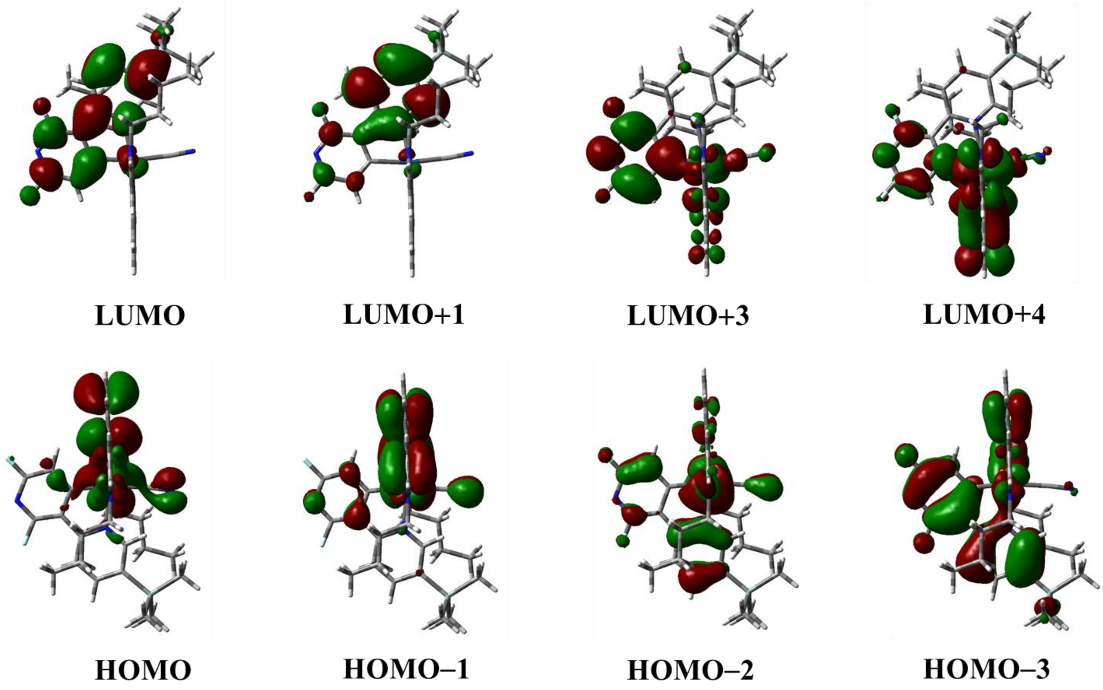

Table S7. The major excitations contributing to the T_1 states of **B–4–TMS** and **B–5–TMS** optimized with the TDDFT/PBE0 method.

Complex	Excitations contributing to the T_1 state	Character
**B–4–TMS**	HOMO–2→LUMO (51.7%)	^{3}LLCT/^{3}MLCT
	HOMO–1→LUMO (22.4%)	^{3}LLCT/^{3}MLCT
	HOMO–3→LUMO (13.8%)	^{3}IL/^{3}LLCT/^{3}MLCL
**B–5–TMS**	HOMO–2→LUMO (51.7%)	^{3}LLCT/^{3}MLCT
	HOMO–1→LUMO (22.4%)	^{3}LLCT/^{3}MLCT
	HOMO–3→LUMO (13.8%)	^{3}IL/^{3}LLCT/^{3}MLCL

Figure S31. Spatial plots (isovalue = 0.02) of selected molecular orbitals of **B–4–TMS** at the optimized T_1 state geometry.

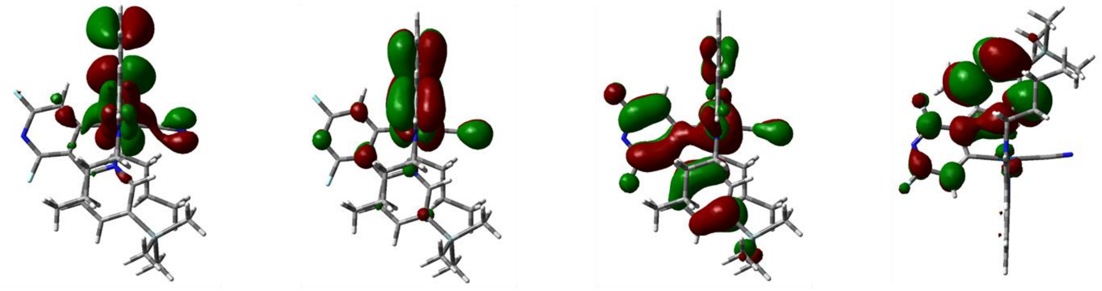

HOMO–1 HOMO–2 HOMO–3 LUMO

Figure S32. Spatial plots (isovalue = 0.02) of selected molecular orbitals of **B–5–TMS** at the optimized T_1 state geometry.

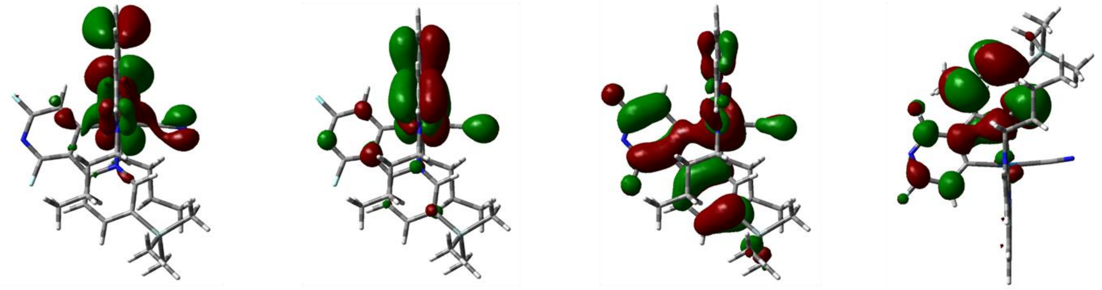

HOMO–1 HOMO–2 HOMO–3 LUMO

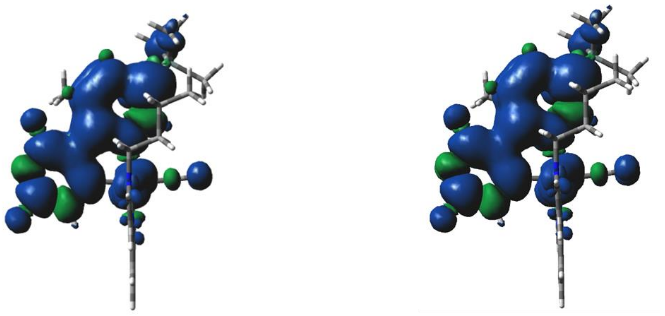

B−4−TMS **B−5−TMS**

Figure S33. Plots of spin density (isovalue = 0.002) of the T_1 states of the two complexes optimized at the PBE0 level.

Table S8. Cartesian coordinates for optimized structures of **B–4–TMS**.

Ground state of **B–4–TMS** (S_0)				Excited state of **B–4–TMS** (T_1)			
Ir	0.879002	0.365093	-0.43483	Ir	0.879002	0.365093	-0.43483
Si	-5.09044	0.075383	-0.90153	Si	-5.09044	0.075383	-0.90153
F	3.873351	-2.24258	3.32594	F	3.873351	-2.24258	3.32594
F	-0.58199	-2.63157	3.723908	F	-0.58199	-2.63157	3.723908
N	2.690415	-1.14804	-2.14821	N	2.690415	-1.14804	-2.14821
N	0.858058	-2.28155	-2.29181	N	0.858058	-2.28155	-2.29181
C	2.766403	0.778266	-0.86878	C	2.766403	0.778266	-0.86878
N	2.486932	2.591433	0.541206	N	2.486932	2.591433	0.541206
C	1.323276	-0.76192	1.216948	C	1.323276	-0.76192	1.216948
N	1.634588	-2.40435	3.503837	N	1.634588	-2.40435	3.503837
C	1.382428	-1.18353	-1.70533	C	1.382428	-1.18353	-1.70533
N	0.580212	2.938868	1.493466	N	0.580212	2.938868	1.493466
C	0.192447	-1.25914	1.934573	C	0.192447	-1.25914	1.934573
C	1.205724	2.088017	0.652816	C	1.205724	2.088017	0.652816
C	3.497703	-0.07061	-1.69495	C	3.497703	-0.07061	-1.69495
C	3.392088	1.879282	-0.29076	C	3.392088	1.879282	-0.29076
N	-0.3741	2.066495	-2.87441	N	-0.3741	2.066495	-2.87441
C	2.590315	-1.10505	1.709374	C	2.590315	-1.10505	1.709374
H	3.499401	-0.7653	1.227485	H	3.499401	-0.7653	1.227485
C	-0.84317	2.859546	1.824576	C	-0.84317	2.859546	1.824576
H	-0.97935	3.323357	2.805788	H	-0.97935	3.323357	2.805788
H	-1.10052	1.80304	1.913098	H	-1.10052	1.80304	1.913098
C	-0.49694	-2.76612	-2.02866	C	-0.49694	-2.76612	-2.02866
H	-0.83243	-3.31917	-2.91045	H	-0.83243	-3.31917	-2.91045
H	-1.13477	-1.88752	-1.91804	H	-1.13477	-1.88752	-1.91804
C	0.129216	1.457211	-2.00821	C	0.129216	1.457211	-2.00821
C	2.961158	-2.21871	-2.98784	C	2.961158	-2.21871	-2.98784
H	3.92347	-2.36935	-3.44918	H	3.92347	-2.36935	-3.44918
C	2.665294	-1.90627	2.836248	C	2.665294	-1.90627	2.836248
C	0.447568	-2.07621	3.039582	C	0.447568	-2.07621	3.039582
C	-3.52097	-0.25021	0.102455	C	-3.52097	-0.25021	0.102455
C	-3.54698	-0.92456	1.335918	C	-3.54698	-0.92456	1.335918
H	-4.49417	-1.21182	1.785549	H	-4.49417	-1.21182	1.785549
C	-2.3692	-1.25468	1.992197	C	-2.3692	-1.25468	1.992197
H	-2.39486	-1.78253	2.933391	H	-2.39486	-1.78253	2.933391
C	-1.1347	-0.91757	1.416263	C	-1.1347	-0.91757	1.416263
N	-1.10732	-0.20849	0.251527	N	-1.10732	-0.20849	0.251527
C	-2.25714	0.101686	-0.37362	C	-2.25714	0.101686	-0.37362
H	-2.12967	0.656668	-1.29544	H	-2.12967	0.656668	-1.29544
C	1.809422	-2.93532	-3.07353	C	1.809422	-2.93532	-3.07353
H	1.576007	-3.83551	-3.61956	H	1.576007	-3.83551	-3.61956
C	-1.7076	3.546155	0.767061	C	-1.7076	3.546155	0.767061
H	-1.38836	4.592819	0.674415	H	-1.38836	4.592819	0.674415
H	-1.52074	3.071025	-0.20342	H	-1.52074	3.071025	-0.20342
C	4.844836	0.167566	-1.96735	C	4.844836	0.167566	-1.96735
H	5.424645	-0.48819	-2.6095	H	5.424645	-0.48819	-2.6095
C	5.44579	1.291072	-1.37891	C	5.44579	1.291072	-1.37891
H	6.492942	1.49311	-1.58067	H	6.492942	1.49311	-1.58067
C	2.636323	3.743959	1.298473	C	2.636323	3.743959	1.298473
H	3.56377	4.290352	1.352234	H	3.56377	4.290352	1.352234
C	-0.55337	-3.63159	-0.76903	C	-0.55337	-3.63159	-0.76903
H	-0.07222	-3.08087	0.045857	H	-0.07222	-3.08087	0.045857
H	0.037355	-4.54375	-0.92561	H	0.037355	-4.54375	-0.92561
C	4.736787	2.158667	-0.53371	C	4.736787	2.158667	-0.53371
H	5.234575	3.014125	-0.08772	H	5.234575	3.014125	-0.08772
C	1.434796	3.966037	1.893417	C	1.434796	3.966037	1.893417
H	1.110761	4.747126	2.562674	H	1.110761	4.747126	2.562674
C	-1.98765	-3.98998	-0.37148	C	-1.98765	-3.98998	-0.37148
H	-2.45061	-4.59546	-1.16258	H	-2.45061	-4.59546	-1.16258

H	-2.58075	-3.06993	-0.29838	H	-2.58075	-3.06993	-0.29838
C	-6.32005	1.020052	0.172244	C	-6.32005	1.020052	0.172244
H	-6.54472	0.47103	1.094275	H	-6.54472	0.47103	1.094275
H	-7.2644	1.170636	-0.36429	H	-7.2644	1.170636	-0.36429
H	-5.93099	2.004135	0.452822	H	-5.93099	2.004135	0.452822
C	-3.19809	3.487604	1.110726	C	-3.19809	3.487604	1.110726
H	-3.3597	3.907716	2.113085	H	-3.3597	3.907716	2.113085
H	-3.51713	2.438374	1.159067	H	-3.51713	2.438374	1.159067
C	-2.0487	-4.74097	0.961095	C	-2.0487	-4.74097	0.961095
H	-1.48757	-5.68182	0.909856	H	-1.48757	-5.68182	0.909856
H	-3.08234	-4.98069	1.234285	H	-3.08234	-4.98069	1.234285
H	-1.61899	-4.14098	1.771265	H	-1.61899	-4.14098	1.771265
C	-4.05507	4.245922	0.094546	C	-4.05507	4.245922	0.094546
H	-3.92927	3.83244	-0.91258	H	-3.92927	3.83244	-0.91258
H	-3.77138	5.304294	0.055104	H	-3.77138	5.304294	0.055104
H	-5.11951	4.194268	0.3483	H	-5.11951	4.194268	0.3483
C	-4.63384	1.045317	-2.4502	C	-4.63384	1.045317	-2.4502
H	-4.14895	1.997443	-2.20966	H	-4.14895	1.997443	-2.20966
H	-5.53288	1.267887	-3.0368	H	-5.53288	1.267887	-3.0368
H	-3.95104	0.476243	-3.09162	H	-3.95104	0.476243	-3.09162
C	-5.80356	-1.61033	-1.36509	C	-5.80356	-1.61033	-1.36509
H	-6.039	-2.19903	-0.47061	H	-6.039	-2.19903	-0.47061
H	-5.08883	-2.18519	-1.96544	H	-5.08883	-2.18519	-1.96544
H	-6.72589	-1.50126	-1.94802	H	-6.72589	-1.50126	-1.94802

Table S9. Cartesian coordinates for optimized structures of **B–5–TMS**.

Ground state of **B–5–TMS** (S_0)				Excited state of **B–5–TMS** (T_1)			
Ir	-0.87894	-0.36506	-0.43504	Ir	0.874754	0.383734	-0.40373
Si	5.09051	-0.07545	-0.9017	Si	-5.1068	0.047874	-0.97228
F	-3.87326	2.24174	3.32637	F	3.761155	-2.00774	3.621054
F	0.58208	2.6307	3.72436	F	-0.64439	-2.92474	3.547016
N	-2.69024	1.14822	-2.14841	N	2.7009	-1.10444	-2.14371
N	-0.8579	2.28179	-2.29176	N	0.931714	-2.34411	-2.19837
C	-2.76635	-0.77814	-0.86905	C	2.752389	0.829936	-0.87343
N	-2.48706	-2.59121	0.54109	N	2.456365	2.678705	0.484159
C	-1.32321	0.76173	1.21691	C	1.302257	-0.7352	1.275939
N	-1.6345	2.40349	3.50427	N	1.541519	-2.4269	3.574124
C	-1.38229	1.18371	-1.70542	C	1.412669	-1.19874	-1.6543
N	-0.58045	-2.93861	1.49358	N	0.550545	3.042926	1.436451
C	-0.19236	1.25876	1.93464	C	0.115532	-1.33609	1.901146
C	-1.2058	-2.08791	0.65265	C	1.187506	2.154935	0.63632
C	-3.49758	0.0708	-1.69522	C	3.481368	0.007158	-1.72648
C	-3.39215	-1.87905	-0.29094	C	3.359327	1.96783	-0.35049
N	0.37408	-2.06644	-2.87467	N	-0.43932	2.079098	-2.82127
C	-2.59024	1.10471	1.70944	C	2.520286	-0.97576	1.88821
H	-3.49933	0.76506	1.22749	H	3.443809	-0.55547	1.504679
C	0.84291	-2.85938	1.82478	C	-0.87077	2.98777	1.787373
H	0.97899	-3.32318	2.80601	H	-0.9684	3.312511	2.829879
H	1.10034	-1.80289	1.91332	H	-1.17307	1.94103	1.732414
C	0.49704	2.76643	-2.02845	C	-0.37365	-2.93712	-1.8949
H	0.83252	3.31967	-2.91012	H	-0.76682	-3.36934	-2.82211
H	1.13496	1.88787	-1.91794	H	-1.03359	-2.1205	-1.60039
C	-0.12918	-1.45713	-2.00846	C	0.109092	1.469034	-1.98498
C	-2.96093	2.21892	-2.98802	C	2.999277	-2.16995	-2.97508
H	-3.92321	2.36956	-3.44941	H	3.950695	-2.27586	-3.471
C	-2.66521	1.90565	2.83652	C	2.577004	-1.79535	3.027982
C	-0.44748	2.07545	3.03993	C	0.383162	-2.21508	3.022362
C	3.52102	0.2502	0.10224	C	-3.54618	-0.33752	0.014389
C	3.54706	0.92436	1.3358	C	-3.58435	-1.12079	1.236008
H	4.49425	1.21155	1.78547	H	-4.5395	-1.44585	1.640595

C	2.36928	1.25438	1.99216	C	-2.43636	-1.4511	1.888183
H	2.39498	1.78208	2.93343	H	-2.4665	-2.03198	2.799719
C	1.13478	0.91735	1.41621	C	-1.14639	-1.02909	1.383597
N	1.10738	0.20847	0.25135	N	-1.12097	-0.19739	0.206799
C	2.25718	-0.10161	-0.37386	C	-2.26455	0.088566	-0.40564
H	2.1297	-0.65644	-1.29578	H	-2.15308	0.702734	-1.29497
C	-1.80922	2.93559	-3.07353	C	1.889172	-2.95094	-3.00708
H	-1.57577	3.83585	-3.61943	H	1.690252	-3.87081	-3.53472
C	1.70737	-3.54605	0.76733	C	-1.73033	3.855466	0.859795
H	1.38822	-4.59276	0.67483	H	-1.36469	4.891465	0.892774
H	1.52041	-3.07107	-0.2032	H	-1.59513	3.50736	-0.17195
C	-4.84476	-0.16722	-1.96752	C	4.802201	0.297684	-2.06775
H	-5.42453	0.48861	-2.60964	H	5.378205	-0.33961	-2.73275
C	-5.44582	-1.29063	-1.37902	C	5.385199	1.451575	-1.52415
H	-6.49301	-1.49255	-1.58073	H	6.412426	1.69397	-1.77884
C	-2.63666	-3.74348	1.2987	C	2.59013	3.869827	1.176838
H	-3.56418	-4.28973	1.35254	H	3.507024	4.436759	1.193153
C	0.55327	3.63173	-0.76869	C	-0.2878	-3.99227	-0.78492
H	0.07204	3.08089	0.04607	H	0.168711	-3.52575	0.095731
H	-0.0375	4.54386	-0.92525	H	0.387024	-4.80148	-1.09726
C	-4.73689	-2.15827	-0.53381	C	4.678456	2.300246	-0.66084
H	-5.23477	-3.01365	-0.08776	H	5.158806	3.186958	-0.25695
C	-1.4352	-3.96554	1.8938	C	1.391977	4.101212	1.771823
H	-1.11129	-4.74648	2.56328	H	1.064699	4.91225	2.40366
C	1.98745	3.9902	-0.3709	C	-1.65861	-4.57074	-0.4094
H	2.45045	4.59584	-1.16187	H	-2.07085	-5.12686	-1.26346
H	2.58066	3.07022	-0.29785	H	-2.35681	-3.7472	-0.20892
C	6.32022	-1.01962	0.1724	C	-6.36628	0.901409	0.162341
H	6.54615	-0.46951	1.09347	H	-6.59171	0.29739	1.04954
H	7.26399	-1.17177	-0.36471	H	-7.31141	1.071469	-0.36844
H	5.93056	-2.00298	0.4547	H	-5.99899	1.87477	0.507905
C	3.19787	-3.48729	1.11088	C	-3.21432	3.820984	1.24636
H	3.3596	-3.90707	2.11336	H	-3.33058	4.146803	2.290321
H	3.51683	-2.43802	1.15888	H	-3.57388	2.783387	1.20745
C	2.04825	4.74106	0.96175	C	-1.59099	-5.48034	0.822824
H	1.487	5.68185	0.91053	H	-0.89122	-6.31109	0.669388
H	3.08182	4.9809	1.2351	H	-2.57281	-5.90989	1.051494
H	1.61851	4.14097	1.77182	H	-1.2576	-4.91944	1.703992
C	4.05481	-4.24587	0.09486	C	-4.08105	4.699587	0.337936
H	3.92841	-3.8331	-0.91248	H	-4.00486	4.38202	-0.70876
H	3.7716	-5.30439	0.05622	H	-3.76888	5.749896	0.387276
H	5.11933	-4.19355	0.34813	H	-5.13686	4.651957	0.628056
C	4.63403	-1.04609	-2.44997	C	-4.69398	1.158609	-2.4439
H	4.1493	-1.99818	-2.20896	H	-4.26905	2.119299	-2.13097
H	5.5331	-1.26881	-3.03646	H	-5.6018	1.372952	-3.02147
H	3.95115	-0.47744	-3.09167	H	-3.97256	0.69422	-3.12594
C	5.80344	1.61016	-1.36594	C	-5.85747	-1.58587	-1.58293
H	6.03725	2.1999	-0.47173	H	-6.06743	-2.27074	-0.75254
H	5.08925	2.18401	-1.9679	H	-5.18158	-2.1011	-2.27546
H	6.72666	1.50102	-1.94744	H	-6.80244	-1.4062	-2.11098

Fabrication of the devices

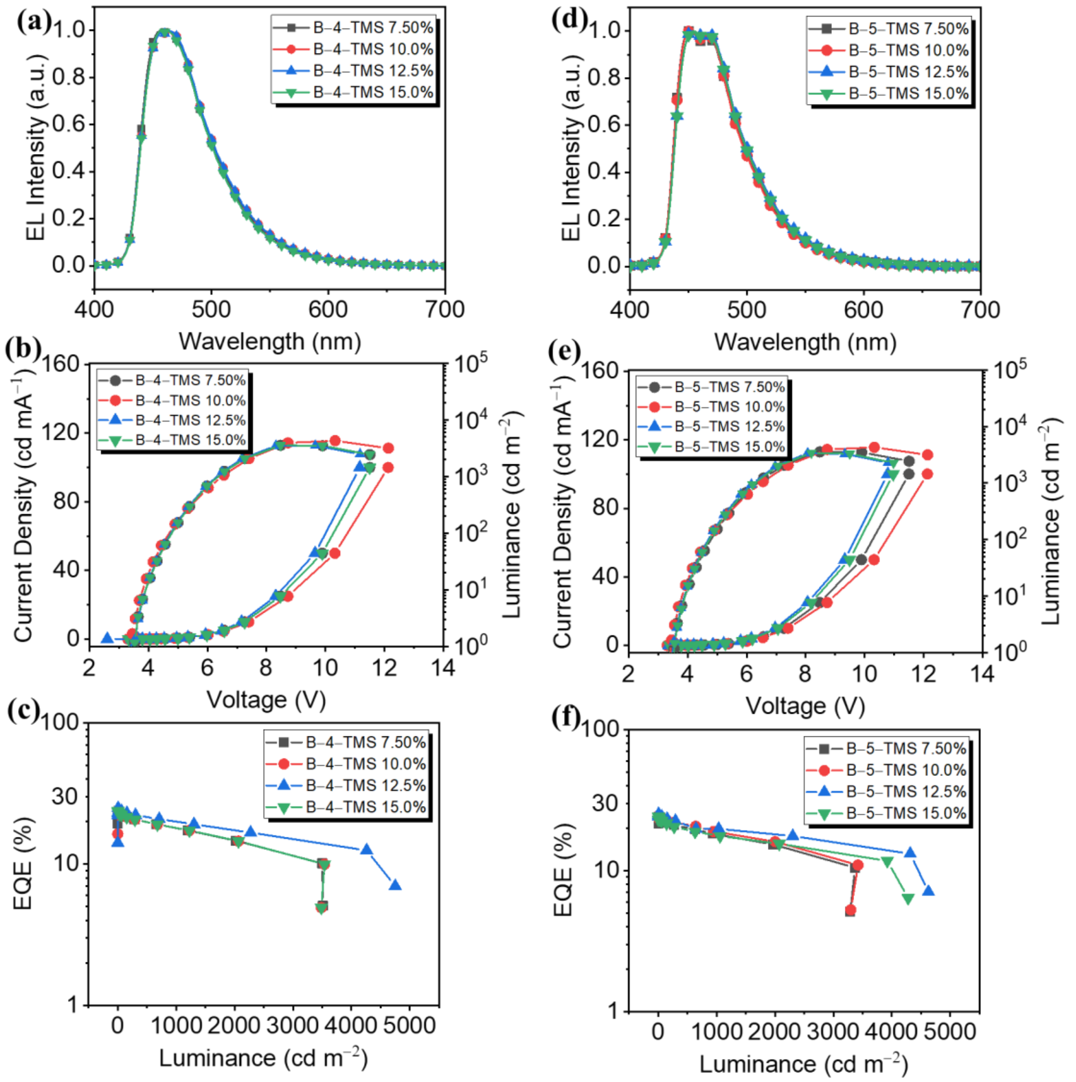

Figure S34. the ratio–dependent phosphorescent device performance of (a) the EL spectrum; (b) the *J*–*V*–*L* carves; (c) the EQE versus luminance for B–4–TMS, and (d) the EL spectrum; (e) the *J*–*V*–*L* carves; (f) the EQE versus luminance for B–5–TMS, respectively.

Table S10. The ratio–dependent EL performances based on the Ph–OLEDs.

Device	λ_{EL} (nm)	L_{max} (cd m^{-2})	EQE[a] (%)	Power efficiency[a] (lm W^{-1})	Current efficiency[a] (cd A^{-1})	CIE (x,y)	FWHM (nm)
B–4–TMS 7.50%	456/463	3510	23.78/22.53/17.56	26.93/21.58/11.61	32.86/31.14/23.99	(0.140,0.174)	63
B–4–TMS 10.0%	456/463	3538	23.91/22.37/7.33	28.71/21.81/11.73	33.88/31.70/24.56	(0.140,0.180)	64
B–4–TMS 12.5%	456/463	4829	25.49/23.85/19.91	29.78/23.43/13.99	34.91/32.67/27.28	(0.140,0.187)	64
B–4–TMS 15.0%	456/463	4750	24.91/23.71/20.86	28.42/23.25/13.13	33.87/32.25/26.10	(0.141,0.181)	62
B–5–TMS 7.50%	450/467	3369	23.94/21.95/18.28	25.67/18.25/11.98	30.77/28.21/23.49	(0.141,0.168)	61
B–5–TMS 10.0%	450/467	3420	23.94/23.78/18.97	25.72/20.77/12.00	29.88/29.69/23.68	(0.141,0.173)	60
B–5–TMS 12.5%	453/467	4471	27.77/22.86/18.91	32.99/22.10/12.78	37.05/30.50/25.23	(0.141,0.174)	62
B–4–TMS 15.0%	453/467	4625	25.20/24.14/19.80	28.37/22.81/13.02	32.97/31.59/25.91	(0.141,0.180)	61

[a.] The value estimated at maximum, 100 cd m^{-2} and 1000 cd m^{-2}.

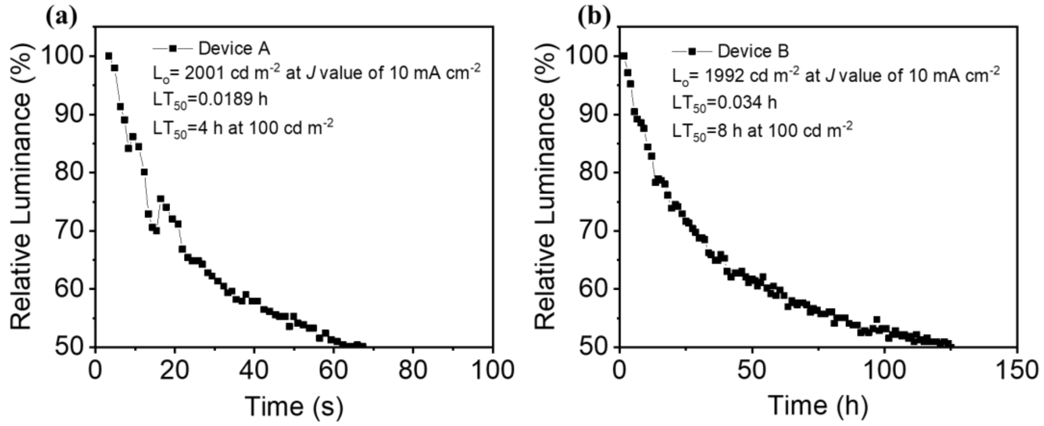

Figure S35. The operational lifetime of device A and B. Device configuration: ITO/HAT−CN (10 nm)/TAPC (40 nm)/TCTA (5 nm)/CzSi (5 nm)/**B–4–TMS** or **B–5–TMS** (12.5%): CzSi (20 nm)/DPEPO (5 nm)/TSPO1 (50 nm)/Liq (2 nm)/Al (120 nm), by assuming an acceleration factor of 1.8.

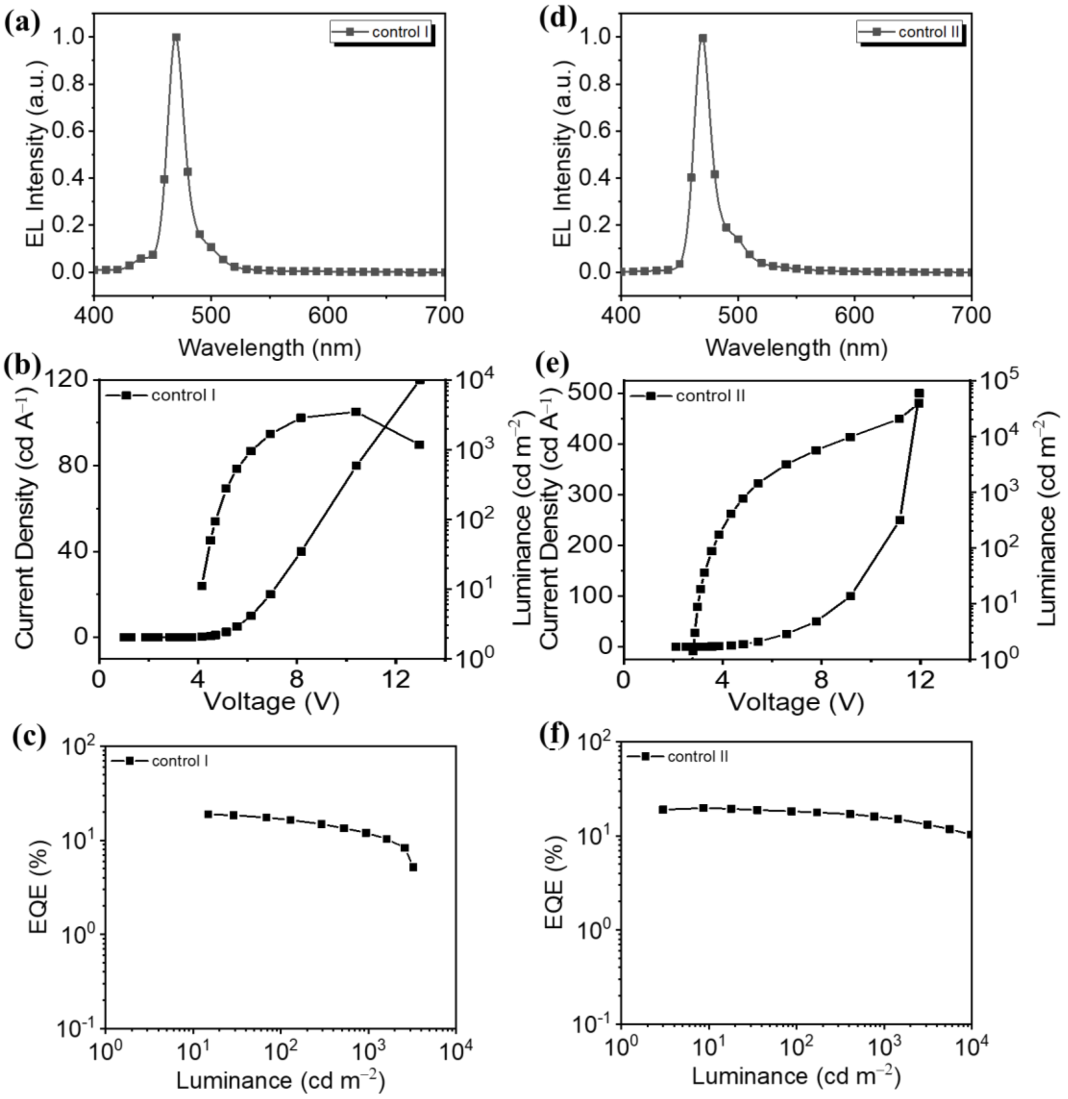

Figure S36. the *v*–DABNA–only device performance of (a) the EL spectrum; (b) the *J–V–L* carves; (c) the EQE versus luminance for **control I**, and (d) the EL spectrum; (e) the *J–V–L* carves; (f) the EQE versus luminance for **control II**, respectively.

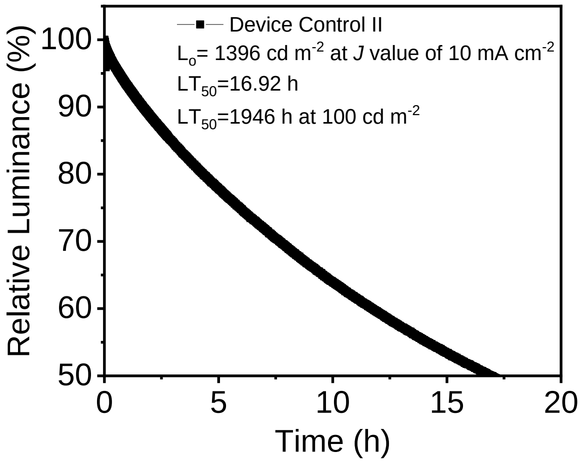

Figure S37. The operational lifetime of device **control II**. Device configuration following literature[26], by assuming an acceleration factor of 1.8.

References

[1] J. Jin, Z. Zhu, J. Yan, X. Zhou, C. Cao, P.-T. Chou, Y.-X. Zhang, Z. Zheng, C.-S. Lee, Y. Chi, *Adv. Photonics Res.* **2022**, 2100381.

[2] C. Wu, M. Wang, K. N. Tong, M. Zhang, W. Li, Z. Xu, W. L. Zhang, Y. Wu, C. Yang, H. Y. Fu, S. S. Chen, M. Ng, M. C. Tang, G. Wei, *Adv. Opt. Mater.* **2022**, 2201998.

[3] K. W. Lo, G. S. M. Tong, G. Cheng, K. H. Low, C. M. Che, *Angew. Chem. Int. Ed.* **2022**, *61*, e202115515.

[4] C.-Y. Chan, M. Tanaka, Y.-T. Lee, Y.-W. Wong, H. Nakanotani, T. Hatakeyama, C. Adachi, *Nat. Photonics* **2021**, *15*, 203-207.

[5] S. Nam, J. W. Kim, H. J. Bae, Y. M. Maruyama, D. Jeong, J. Kim, J. S. Kim, W. J. Son, H. Jeong, J. Lee, S. G. Ihn, H. Choi, *Adv. Sci.* **2021**, *8*, e2100586.

[6] S. O. Jeon, K. H. Lee, J. S. Kim, S.-G. Ihn, Y. S. Chung, J. W. Kim, H. Lee, S. Kim, H. Choi, J. Y. Lee, *Nat. Photonics* **2021**, *15*, 208-215.

[7] W. J. Chung, K. H. Lee, M. Jung, K. M. Lee, H. C. Park, M. S. Eum, J. Y. Lee, *Advanced Optical Materials* **2021**, *9*, 2100203.

[8] X. Yang, X. Zhou, Y. X. Zhang, D. Li, C. Li, C. You, T. C. Chou, S. J. Su, P. T. Chou, Y. Chi, *Adv. Sci. (Weinh)* **2022**, e2201150.

[9] Y. Qin, X. Yang, J. Jin, D. Li, X. Zhou, Z. Zheng, Y. Sun, W. Y. Wong, Y. Chi, S. J. Su, *Adv. Opt. Mater.* **2022**, 2201633.

[10] D. Zhang, X. Song, A. J. Gillett, B. H. Drummond, S. T. E. Jones, G. Li, H. He, M. Cai, D. Credgington, L. Duan, *Adv. Mater.* **2020**, *32*, e1908355.

[11] S. H. Han, J. H. Jeong, J. W. Yoo, J. Y. Lee, *J. Mater. Chem. C* **2019**, *7*, 3082-3089.

[12] K. H. Lee, J. Y. Lee, *J. Mater. Chem. C.* **2019**, *7*, 8562-8568.

[13] H. J. Cheon, S. J. Woo, S. H. Baek, J. H. Lee, Y. H. Kim, *Adv. Mater.* **2022**, *34*, e2207416.

[14] X. Lv, J. Miao, M. Liu, Q. Peng, C. Zhong, Y. Hu, X. Cao, H. Wu, Y. Yang, C. Zhou, J. Ma, Y. Zou, C. Yang, *Angew Chem Int Ed Engl* **2022**, *61*, e202201588.

[15] K. Stavrou, S. Madayanad Suresh, D. Hall, A. Danos, N. A. Kukhta, A. M. Z. Slawin, S. Warriner, D. Beljonne, Y. Olivier, A. Monkman, E. Zysman-Colman, *Adv. Opt. Mater.* **2022**, *10*, 2200688.

[16] R. Braveenth, H. Lee, J. D. Park, K. J. Yang, S. J. Hwang, K. R. Naveen, R. Lampande, J. H. Kwon, *Adv. Funct. Mater.* **2021**, *31*, 2105805.

[17] J. P. Eungdo Kim, Mieun Jun, Hyosup Shin, Jangyeol Baek, Taeil Kim, Seran Kim, Jiyoung Lee, Heechoon Ahn, Jinwon Sun, Soo-Byung Ko, Seok-Hwan Hwang, Jun Yeob Lee, Changwoong Chu, Sunghan Kim, *Sci. Adv.*, *8*, 9.

[18] H. Lee, R. Braveenth, S. Muruganantham, C. Y. Jeon, H. S. Lee, J. H. Kwon, *Nat. Commun.* **2.023**, *14*, 419.

[19] Z. L. Zhu, P. Gnanasekaran, J. Yan, Z. Zheng, C. S. Lee, Y. Chi, X. Zhou, *Inorg. Chem.* **2022**, *61*, 8898-8908.

[20] M. J. Frisch, G. W. Trucks, H. B. Schlegel, G. E. Scuseria,
M. A. Robb, J. R. Cheeseman, G. Scalmani, V. Barone,

G. A. Petersson, H. Nakatsuji, X. Li, M. Caricato, A. V. Marenich, J. Bloino, B. G. Janesko, R. Gomperts, B. Mennucci, H. P. Hratchian, J. V. Ortiz, A. F. Izmaylov, J. L. Sonnenberg, D. Williams-Young, F. Ding, F. Lipparini, F. Egidi, J. Goings, B. Peng, A. Petrone, T. Henderson, D. Ranasinghe, V. G. Zakrzewski, J. Gao, N. Rega, G. Zheng, W. Liang, M. Hada, M. Ehara, K. Toyota, R. Fukuda, J. Hasegawa, M. Ishida, T. Nakajima, Y. Honda, O. Kitao, H. Nakai, T. Vreven, K. Throssell, J. A. Montgomery, Jr., J. E. Peralta, F. Ogliaro, M. J. Bearpark, J. J. Heyd, E. N. Brothers, K. N. Kudin, V. N. Staroverov, T. A. Keith, R. Kobayashi, J. Normand, K. Raghavachari, A. P. Rendell, J. C. Burant, S. S. Iyengar, J. Tomasi, M. Cossi, J. M. Millam, M. Klene, C. Adamo, R. Cammi, J. W. Ochterski, R. L. Martin, K. Morokuma, O. Farkas, J. B. Foresman, and D. J. Fox, Gaussian, Inc., Wallingford CT, 2019.

[21] C. Adamo and V. Barone, J. Chem. Phys., 1999, 110, 6158.

[22] P. J. Hay and W. R. Wadt, J. Chem. Phys., 1985, 82, 270.

[23] P. J. Hay and W. R. Wadt, J. Chem. Phys., 1985, 82, 299.

[24] O. Tapia, J. Math. Chem., 1992, 10, 139.

[25] J. Tomasi and M. Persico, Chem. Rev., 1994, 94, 2027.

[26] S. Jinwon, A. Heechoon, K. Sunwoo, K. Soo-Byung, S. Dayoon, U. Hyun Ah, K. Sungbum, L. Yoonkyoo, J. Pyungeun, H. Seok-Hwan, Y. Youngmin, C. Changwoong, K. Sunghan, *Nat. Photonics* **2022**, *16*, 212-218.
